# RNA editing is abundant and correlates with task performance in a social bumblebee

**DOI:** 10.1038/s41467-019-09543-w

**Published:** 2019-04-08

**Authors:** Hagit T. Porath, Esther Hazan, Hagai Shpigler, Mira Cohen, Mark Band, Yehuda Ben-Shahar, Erez Y. Levanon, Eli Eisenberg, Guy Bloch

**Affiliations:** 1 The Mina and Everard Goodman Faculty of Life Sciences, Bar Ilan University, Ramat-Gan, 52900 Israel; 2 Department of Ecology, Evolution & Behavior, Alexander Silberman Institute of Life Sciences, Hebrew University of Jerusalem, Jerusalem, 91904 Israel; 3 Roy J Carver Biotechnology Center, The University of Illinois at Urbana-Champaign, Champaign, 61801 IL USA; 4 Institute of Evolution, University of Haifa, Haifa, 3498838 Israel; 5 Department of Biology, Washington University in St. Louis, St. Louis, 63130-4899 MO USA; 6 Raymond and Beverly Sackler School of Physics and Astronomy and Sagol School of Neuroscience, Tel Aviv University, Tel Aviv, 6997801 Israel

## Abstract

Colonies of the bumblebee *Bombus terrestris* are characterized by wide phenotypic variability among genetically similar full-sister workers, suggesting a major role for epigenetic processes. Here, we report a high level of ADAR-mediated RNA editing in the bumblebee, despite the lack of an ADAR1-homolog. We identify 1.15 million unique genomic sites, and 164 recoding sites residing in 100 protein coding genes, including ion channels, transporters, and receptors predicted to affect brain function and behavior. Some edited sites are similarly edited in other insects, cephalopods and even mammals. The global editing level of protein coding and non-coding transcripts weakly correlates with task performance (brood care vs. foraging), but not affected by dominance rank or juvenile hormone known to influence physiology and behavior. Taken together, our findings show that brain editing levels are high in naturally behaving bees, and may be regulated by relatively short-term effects associated with brood care or foraging activities.

## Abstract (web summary)

Bumblebee workers are genetically highly similar but they show different behaviors such as brood care and foraging. Here the authors report a high level of ADAR-mediated RNA editing in the bumblebee *Bombus terrestris* and its weak correlation to task performance.

## Introduction

Social insects such as ants, termites, wasps and bees, provide excellent model systems for studying how a single genome can generate substantial functional diversity. Individual social insects typically show remarkable plasticity, switching between behavioral and physiological states along with the social environment they experience, during development, or even as fully formed adult insects^1,2^. Variation in behavioral and physiological state in many species is not associated with genetic variation (which is often fairly low), but rather with differential expression of transcription factors, histone acetylation, DNA methylation, and non-coding RNA regulation^3–5^. Thus, much attention has been given to epigenetic regulatory processes that may contribute to the regulation of gene expression and increase the functional diversity among genetically similar individuals. These processes include chemical alterations of DNA, chromatin modifiers, non-protein coding RNAs, as well as modifications to the sequence or chemistry of coding and non-coding RNAs^3^. Previous studies have indeed suggested that methylation mediates changes in brain function in response to even small changes in the environment or  in social role^6,7^. However, the rich behavioral repertoire of social insects is complex and cannot be fully accounted for by methylation or any other of the abovementioned processes alone^8^.

Here, we study RNA editing, an epigenetic process that modifies nucleotides in pre-mRNA sequences in a social bumblebee. We focus on RNA editing, mediated by the ADAR (adenosine deaminase acting on RNA) enzymes, the most common type of editing known in animals. ADAR enzymes are evolutionary conserved, predominantly active in the nervous system, and act through selective deamination of specific adenosines to inosines (“A-to-I editing”) within the target RNA^9,10^. Three ADAR enzymes are encoded by the mammalian genome: double-stranded RNA (dsRNA)-specific adenosine deaminase (ADAR1, or ADAR), dsRNA-specific editase 1 (ADAR2, or ADARB1) and the catalytically inactive ADAR3 (or ADARB2). Insects are believed to encode a single ADAR enzyme, orthologous to mammalian ADAR2^11^. Given that inosine is read as guanosine by the translation machinery, RNA editing can lead to changes in protein sequences and function^12^. A-to-I editing of protein coding genes may contribute to behavioral plasticity^12^ by modifying the amino acid sequence (“recoding”), altering splice sites^13^, or affecting the regulation of gene expression. Recoding of even a single amino acid may affect protein function. For example, modifications in the catalytic site of an enzyme in the pore forming regions of an ion channel, can profoundly affect specificity, level of activity or ion permeability^14,15^. ADAR can also affect gene expression patterns by editing non-coding RNA such as microRNAs and long non-coding RNAs^16,17^. The fraction of edited molecules among the RNA copies of a given editing target varies between 0–100% and may be tissue or even cell-dependent^18^. Editing levels are dynamically regulated, and were shown to depend on developmental stages, various disease states, as well as environmental conditions^19^. Thus, RNA editing is well positioned to increase phenotypic variability by providing functional heterogeneity across tissues, brain regions or even among cells within the same tissue. The temporal regulation of RNA editing and its sensitivity to varying conditions and environments (developmental stages, senescence, or experience) may contribute to behavioral or developmental plasticity.

Given the broad spectrum of ADAR activity, it is not surprising that A-to-I RNA editing has been shown to affect physiology and behavior (for recent reviews see refs. ^10,20^). For example, in the nematode *Caenorhabditis elegans* ADAR mutants show impaired chemotaxis and abnormal behavior^21^. In the fruit fly *Drosophila melanogaster*, ADAR mutants are morphologically normal but exhibit extreme behavioral deficits that include temperature-sensitive paralysis, impaired locomotion coordination, abnormal courtship behavior, weaker circadian rhythms, and aberrant sleep patterns^22–24^. Mice mutants with homozygotic deletions of ADAR2 show frequent seizures and die prematurely. Given the substantial effects on behavior and physiology, and its dynamic regulation, we wondered whether RNA editing also plays a role in the social regulation of behavior, a noted hallmark of insect societies. The importance of RNA editing in social insects has so far only been investigated in the leaf-cutting ant (*Acromyrmex echinatior*), which lives in complex societies with morphologically distinct castes. Approximately 800 transcripts expressed in the ant head showed evidence of A-to-I RNA editing. These transcripts were functionally enriched for genes involved in neurotransmission, circadian rhythms, temperature response, RNA splicing and carboxylic acid biosynthesis. Some editing sites were conserved across ant subfamilies. Moreover, the level of RNA editing of some transcripts was associated with caste differentiation^25^. Caste differentiation in leaf-cutting ants occurs early in development and produces very distinct queen and worker phenotypes that differ greatly in their behavior and physiology. It remains unknown whether RNA editing also contributes to dynamic, more subtle, changes in behavior of adult social insects.

The bumblebee *Bombus terrestris* provides an excellent model system with which to study dynamic A-to-I RNA editing in an ecological context. The bumblebee queen typically mates with a single haploid male producing a family (colony) of genetically highly related “full-sister” worker bees (genetic relatedness, *r* = 0.75). Although genetic variation among full-sisters is low, they are yet remarkably diverse in terms of body size, physiology, reproductive state, sensory sensitivity, circadian rhythmicity, learning capacity, and behavior (e.g., refs. ^26–29^). Unlike the caste system of ants, task performance of bumblebee workers is much less rigid over time, and individual bees commonly switch between brood care and foraging activities within the same day^28^. Here, we study the association between RNA editing and behavioral variation among genetically related bumblebee workers. Combining RNA sequencing and behavioral observations, we show that A-to-I RNA editing in the bumblebee brain is extensive, and weakly correlates with task performance.

## Results

### The *B. terrestris* genome encodes a single ADAR enzyme

The RefSeq protein database reports two ADAR variants that are both mapped to a single locus in the *B. terrestris* genome. These predicted proteins are close homologs of the Drosophila ADAR and the mammalian ADAR2. We verified that the available *B. terrestris* genome (see Methods) does not encode for additional ADAR genes. We also found two ADAR splice variants in the honeybee *A. mellifera* (Supplementary Fig. 1B and Supplementary Note 1). Given that we were specifically interested in testing the relationship between RNA editing and behavior and that BtADAR is enriched in the brain (see below), we further focused on RNA editing and BtADAR expression in the whole brain tissue.

### A-to-I RNA hyper-editing events

A-to-I editing sites often appear in clusters, but some sites, mainly those within the coding sequence, are commonly isolated. We therefore used two complementary methods. The first is set to detect heavily (hyper) edited reads supporting clusters of editing sites, and the second takes advantage of all RNA-seq reads aligned to a genomic location in search of isolated editing events.

**Fig. 1:**
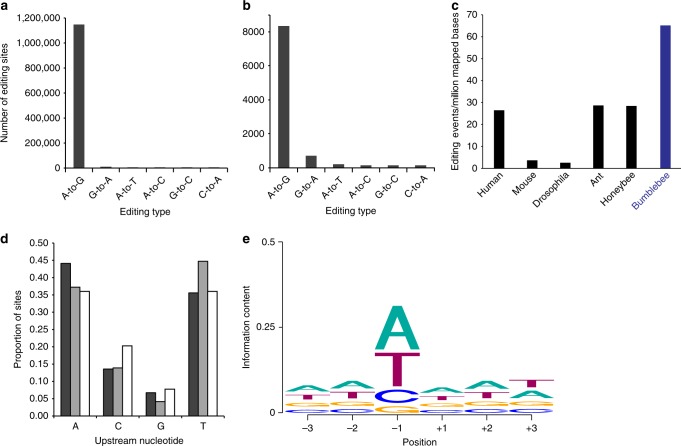
RNA editing is abundant in the *Bombus terrestris* brain. **a**, **b** Two complementary RNA editing detection schemes result in a strong and clean signal: **a** A hyper-editing pipeline detecting clusters of putative editing sites on RNA samples. **b** The MuTect pipeline that compares the RNA samples to a DNA sequence compiled from sequencing the genome of four representative bee samples. The analyses were performed on a brain RNA-seq dataset obtained from 58 bumblebee workers. Both methods identified many A-to-G mismatch sites (likely due to A-to-I editing), with high specificity. Specificity is assessed by applying the same procedure to all mismatch types and looking at the ratio of A-to-G to non-A-to-G mismatches. **c** Normalized hyper-editing signal (A-to-G mismatches detected in clusters, per million mapped bases) in the bumblebee brain, compared with human brain, mouse brain, drosophila head^33^, honeybee brain^6^, and leaf-cutting ant head^25^. **d**, **e** Sequence motif for predicted RNA editing sites. **d** Distribution of the nucleotide upstream to the predicted editing sites for sites detected by the hyper-editing (dark gray bars) and MuTect pipelines (light gray bars) in the current study, compared with the one observed in a previous study with *Drosophila* (open bars,^37^). In all three cases, G is depleted in the base upstream to the predicted editing site. **e** Base preference around the detected hyper-editing sites presented by WebLogo. Position 0 is the edited A (not presented), positions −3, −2, −1 and 3, 2, 1 are the upstream and downstream bases, respectively). “G” is depleted in the nucleotide upstream to the editing site, and seen in only ~7% of the sites. The downstream nucleotide is enriched for “A” (34%), in accordance with the ADAR motif observed in amphibians and invertebrates^33^. Source data are provided as a Source Data file

To find clustered editing sites, we applied the hyper-editing detection scheme^30^. This approach looks for multiple consecutive mismatches of the same type between the reference genome and the RNA-seq data (e.g., genomic-A: RNA-G), which are unlikely to result from genomic polymorphisms^12^. We analyzed RNA-seq data of brain samples originating from 58 individual bumblebee workers, consisting of ~4 billion 2 × 100 bp reads. Among these, we identified 4,040,095 hyper-edited reads harboring 24,314,377 editing events, located at 1,150,394 unique genomic sites that cluster to 51,930 distinct genomic regions (see Methods). Specificity was very high, with noise level lower than 1% (Fig. 1a). On average, an RNA edited cluster was 128bp ± 118 (1 std) long and contained 22 ± 29 (1 std) editing sites (full distributions are presented in Supplementary Fig. 2). Thus, the majority of adenosines in these regions may be edited.

The sequence context of the putative editing sites is consistent with the known preference of ADARs (both ADAR1 and ADAR2) in metazoans^31–33^ (Fig. 1d, e). Moreover, the hyper-edited regions are enriched in putative long, stable, dsRNA structures: 20% of the regions (916/4510) show a reverse-complement alignment within their flanking ( ± 2 kb) genomic sequence ( ≥ 65% identity along ≥ 60% of the region; see Methods), compared with only 3.3% (147/4510; *P*-value < 1e-100, *χ*^2^-test) for the control (alignment to the same-strand using the same parameters). Furthermore, putative dsRNAs were found for only 1.6% (73/4510) of random regions of similar length. Taken together, the remarkably high signal-to-noise ratio, the depletion of G in the -1 position, and the enrichment of dsRNA structures, strongly support the premise that the sites detected represent genuine A-to-I editing sites.

Notably, the normalized hyper-editing signal (total A-to-G mismatches detected per million mapped bases) for *B. terrestris* brain (65.1) was ~30-fold higher than in the fruit fly *(Drosophila melanogaster*) head^33^ (2.57), the honeybee (*A. mellifera*) brain^6^ (28.5), the leaf-cutting ant *(Acromyrmex echinatior*) head^25^ (28.7), and the human brain^33^ (26.5) (Fig. 1c). Generally, widespread editing is considered to be carried out by ADAR1^19^, which is thought to be lost in insects^11^. Our results for the bumblebee, nevertheless, demonstrate that abundant hyper-editing may be present even in insects lacking ADAR1 (Fig. 1c).

While the magnitude of hyper-editing is impressive, one expects much of the functional impact of differential editing to come from sites in coding regions. Among the sites identified by the hyper-editing method, we found 208,089 sites (18%, Supplementary Data 1) residing in annotated coding regions (NCBI RefSeq annotations, see Methods), but only a minute fraction of these are well-expressed and edited to a high level. Profiling the hyper-editing level in protein coding sites across the 58 samples, we found that only 253 (0.12%) have a median coverage of at least 50 reads and a median editing level exceeding 5%. Furthermore, analysis of editing level distribution reveals that the majority of these sites are actually genomic polymorphisms, rather than editing sites (see Supplementary Note 2). Therefore, in order to find a set of bona-fide highly edited sites within coding regions, sites that are often not a part of a large cluster, we used a complementary detection method comparing RNA and DNA sequences.

### Detection of single editing sites from DNA–RNA mismatches

We sequenced the DNA of four of the 58 bees, aligned the matched DNA and RNA data to the genome, and used the MuTect tool^34^ to identify 9653 reliable DNA–RNA mismatches. This method is less sensitive to clusters of editing sites, as it misses many clusters due to low DNA-seq coverage, for example. However, it allows for detection of editing sites that are not part of a cluster, and results in a set of editing sites that are (on average) more highly expressed and more highly edited than those found by the hyper-editing scheme. The genetic background of the four bees was relatively homogenous as expected from their genetic relatedness (see Supplementary Methods and Supplementary Fig. 3). Out of the six possible mismatches types, 86% (8340, Supplementary Data 2) were A-to-G editing sites, with the next common mismatch (G-to-A) accounting for only 7% of the sites (see Fig. 1b). Here too, G was strongly depleted (4.2%) upstream of the predicted sites (Fig. 1d). The majority of A-to-G sites identified by this method (4872/8340; 58%) were also detected by the hyper-editing pipeline. Among these, 219 sites reside in protein coding regions (Supplementary Data 3), 63 (29%), of which were also detected by the hyper-editing method. Notably, the fraction of A-to-G sites among all mismatches identified by MuTect in coding regions was 64% (219/341), providing a signal-to-noise ratio much better than that obtained by standard RNA editing detection schemes^12^. Three quarters (164/219) of the sites in coding region are non-synonymous (“recoding”) sites. The sites are well-covered (reads per site per sample: average 107 ± 102 (1 std), median 85), and the editing levels (averaged over the 58 bee samples) vary, with an average of 26% ± 27% (1 std) and median of 16%. Editing was overall lower in protein coding sequences (CDS) relative to all other sites (Mann–Whitney test, *P*-value = 4.95E-04 Supplementary Fig. 4). The coding editing sites reside within 100 different genes (Supplementary Data 4), including receptors and many ion channel proteins such as the *Shab*, *Shal*, *Shaw*, and *EAG* potassium voltage-gated channel proteins. GO analysis revealed a highly significant enrichment for ion channels, ion transport and voltage-gated potassium channel complex (Supplementary Data 5). For the analyses below, we focused on 149 well-edited and well-covered sites (median coverage of at least 50 reads and a median editing level that exceeds 5%, Supplementary Data 6).

### Evolutionary conserved recoding sites

**Fig. 2:**
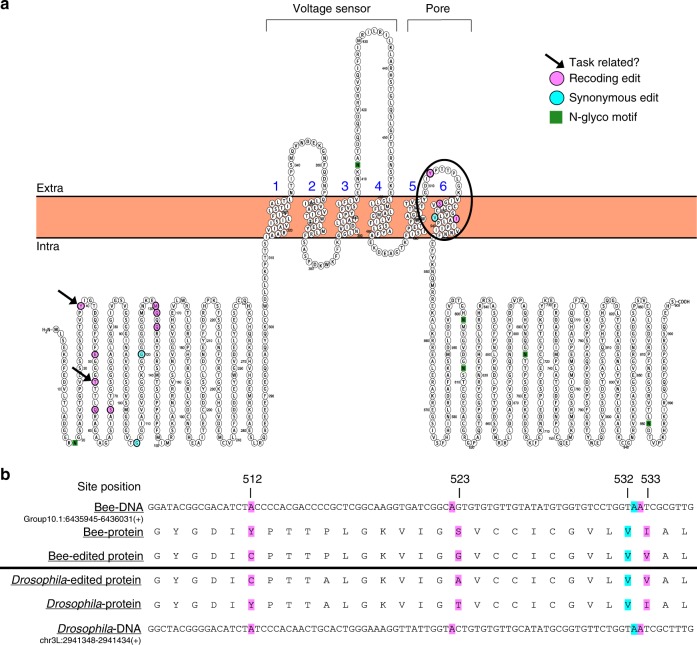
A-to-I RNA editing of the bumblebee *Shab* voltage-gated ion channel transcript. **a** A predicted model of the *Bombus terrestris Shab*-like protein showing the location of edited sites. The arrows point to sites differentially edited between nurses and foragers. Recoding (non-synonymous) and synonymous sites are highlighted in pink and light-blue, respectively. The cluster of four highly conserved editing sites on the 6th transmembrane domain is highlighted with a black circle. These four sites are conserved in other insects, cephalopods (the three recoding sites) and mammals (the last recoding site). **b** Alignment of the *Shab* gene for *Bombus terrestris* (top) and *Drosophila melanogaster*. Predicted conserved RNA editing sites are highlighted (pink–recoding; light-blue-synonymous) (see also Table 2 and Supplementary Fig. 5)

Seven of the 164 recoding sites are also “highly conserved” in Drosophila^35^ (see Methods) and exhibit similar editing levels in both species^36^ (Supplementary Data 7), suggesting they may be conserved across insecta. The bee orthologue of the potassium voltage-gated channel *Shab*, showed in our analyses 11 recoding sites and 3 synonymous sites (Table 1 and Fig. 2) that were clustered on two regions of the protein. Ten editing sites are mapped to the N-terminus inner cytoplasmic domain, and the remaining four on the 6th transmembrance domain (Fig. 2). The latter four sites are strongly edited (93–98% median editing, *n* = 58 bee samples) and conserved in *Drosophila melanogaster* and other insects (refs. ^37,38^; Fig. 2 and Table 2). Three are non-synonymous, and their recoding is also observed in four cephalopod species (two squid species: *Sepia oficianalis*, *Doryteuthis pealeii*, and two octopus species: *Octopus vulgaris* and *O. bimaculoides*^39^; and Table 2). Interestingly, in site Bt Y512C, the squid genome encodes the edited version, as well as in site Bt S523G for the two octopus species (see Table 2 and Supplementary Fig. 5). Remarkably, one of the conserved recoding sites (Bt I533V) is similarly edited in mammals, making it the only known example of a recoding site conserved across metazoa (Supplementary Fig. 5).

**Table 1:** Putative RNA editing sites on the bumblebee *Shab* potassium channel protein

Region	Position	HE^a^	Protein position	Codon change (AA)	Median reads	Median edit
Group10.1	6425707	Yes	39	TAC (Y;Tyr) ⇒ TGC (C;Cys)	101	0.29
Group10.1	6425737	Yes	49	GAG (E;Glu) ⇒ GGG (G;Gly)	100	0.34
Group10.1	6425748	Yes	53	ACG (T;Thr) ⇒ GCG (A;Ala)	86	0.53
Group10.1	6425761	Yes	57	CAG (Q;Gln) ⇒ CGG (R;Arg)	90	0.72
Group10.1	6425805	Yes	72	AGC (S;Ser) ⇒ GGC (G;Gly)	97	0.29
Group10.1	6425912	No	107	Syn: GGA (G;Gly) ⇒ GGG (G;Gly)	31	0.14
Group10.1	6425951	No	120	Syn: GGA (G;Gly) ⇒ GGG (G;Gly)	41	0.09
Group10.1	6426120	No	130	ATG (M;Met) ⇒ GTG (V;Val)	69	0.07
Group10.1	6426124	No	131	CAG (Q;Gln) ⇒ CGG (R;Arg)	70	0.19
Group10.1	6426130	No	133	CAG (Q;Gln) ⇒ CGG (R;Arg)	67	0.35
Group10.1	6435961	Yes	512	TAC (Y;Tyr) ⇒ TGC (C;Cys)	91	0.93
Group10.1	6435993	Yes	523	AGT (S;Ser) ⇒ GGT (G;Gly)	82	0.12
Group10.1	6436022	Yes	532	Syn: GTA (V;Val) ⇒ GTG (V;Val)	90	0.97
Group10.1	6436023	Yes	533	ATC (I;Ile) ⇒ GTC (V;Val)	93	0.98

^a^HE = detected also in the hyper-editing analyses

**Table 2:** Comparison of *Shab* editing in bumblebees, Drosophila, and Cephalopods

Bumblebee					*Drosophila melanogaster*					Cephalopods			
Chr.	Position (strand)	Protein position	AA- change	Editing level	Chr.	Position (strand)	Protein position	AA- change	Editing level	Cuttlefish (*Sepia oficianalis*)	Squid (*Doryteuthis pealeii*)	*Octopus vulgaris*	*Octopus bimaculoides*
Group10.1	6435961 (+)	512	YC	0.93	3L	2941364 (+)	660	YC	0.62	YC	C	YC	YC
Group10.1	6435993 (+)	523	SG	0.12	3L	2941396 (+)	671	TA	0.31	SG	SG	G	G
Group10.1	6436022 (+)	532	VV	0.97	3L	2941425 (+)	680	VV	0.45	Not conserved			
Group10.1	6436023 (+)	533	IV	0.98	3L	2941426 (+)	681	IV	0.77	IV	IV	IV	IV

For additional information see Fig. 2, Supplementary Data 7, and Supplementary Fig. 5.

### Autoediting of BtADAR

**Fig. 3:**
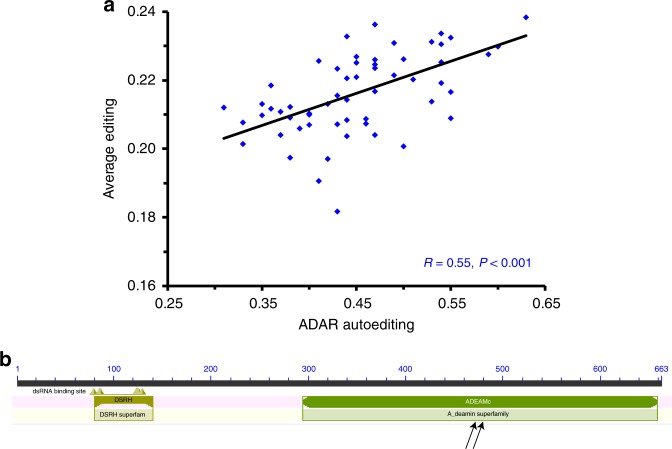
ADAR autoediting is weakly but positively correlated with overall RNA editing. **a** The plot shows Pearson’s correlation between the editing levels at 2609 coding and non-coding sites with a median coverage ≥ 50 reads and the BtADAR autoediting level at site Group15.5:2877069 (position 482 on the protein sequence). Source data are provided as a Source Data file. **b** The arrows point to the location of the two non-synonomous editing sites within the bumblebee ADAR protein

The BtADAR transcript contains two recoding sites (K481E and I482M; Fig. 3b) and one synonymous site at codon 501. Median editing levels at these sites are 7%, 45%, and 3%, respectively. The level of the most highly edited I482M site is positively correlated with the global editing activity (editing index averaging over the 2609 sites detected by MuTect and covered by at least 50 reads, see Methods) (Fig. 3a), and with the editing level of 14 coding sites, including two on ADAR, two on Sodium Channel 60E, and one on *Shab* (Supplementary Data 8). ADAR transcript abundance is not correlated with autoediting level in any of the three sites (*P*-values = 0.47, 0.36, and 0.31, respectively, data not shown).

ADAR autoediting in the bumblebee targets different amino acids than in Drosophila and mice (Supplementary Note 3) in which autoediting was shown to affect global editing activity^13,40,41^. It is thus important to note that the observed correlation between autoediting level at the I482M site and the overall editing activity may be simply explained by an increase in overall editing activity (even though no correlation with ADAR expression is observed) and at this stage, should not be interpreted as a sign for increased editing activity. Yet, we cannot reject the possibility that autoediting modifies the functionality of ADAR and affects the global editing level. This interesting possibility deserves further research.

### Differences in editing level among worker bees

**Fig. 4:**
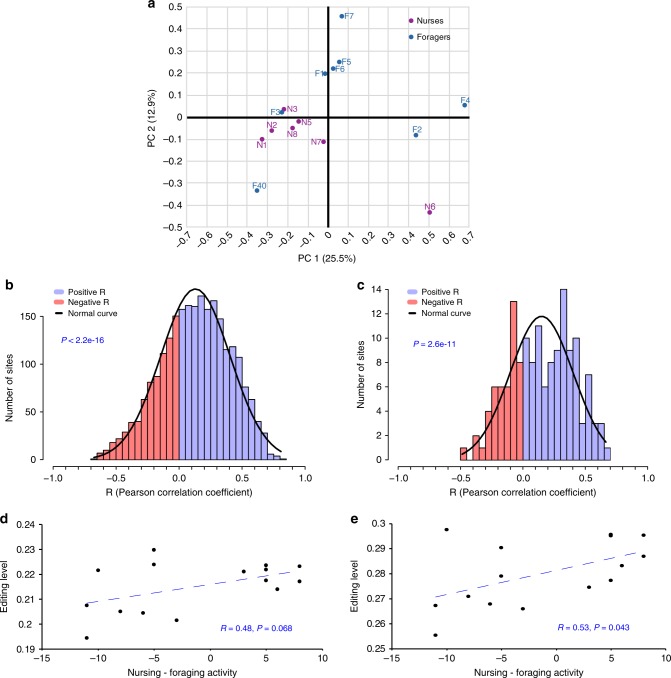
RNA editing correlates with the level of nursing–foraging activity. **a** PCA analysis showing the clustering of bees classified as nurses or foragers based on their RNA editing level profile across 149 well-covered sites within the coding region. **b**, **c** Distribution of the correlation coefficients between the task performance index (the number of observations of nursing activity minus the number of observations of foraging activity) and editing levels at each editing site. Blue and red bars show sites with positive and negative correlation coefficient, respectively. Significant enrichment of positive correlations was observed for sites in non-coding (**b**, 2468 sites), as well as coding (**c**, 149 sites) regions (*t*-test). **d**, **e** Pearson’s correlation between overall editing level (index overall sites, see Methods) and task performance index for non-coding (**d**) and protein coding (**e**) sites. Source data are provided as a Source Data file

To study the correlation between editing and task performance (brood care vs. foraging activities), we observed 15 individual worker bees for 3 successive days, and found 8 of them to perform mostly foraging activities (termed “foragers”), and the other 7 to mostly tend brood (“nurses”, see Methods). We looked at the editing profile for these 15 bees, considering all sites residing in coding regions with a median coverage of 50 reads or more (149 sites, Supplementary Data 6). PCA analysis revealed editing profiles cluster in accordance with task performance, with the exception of two foragers clustering with the nurses (Fig. 4a).

**Fig. 5:**
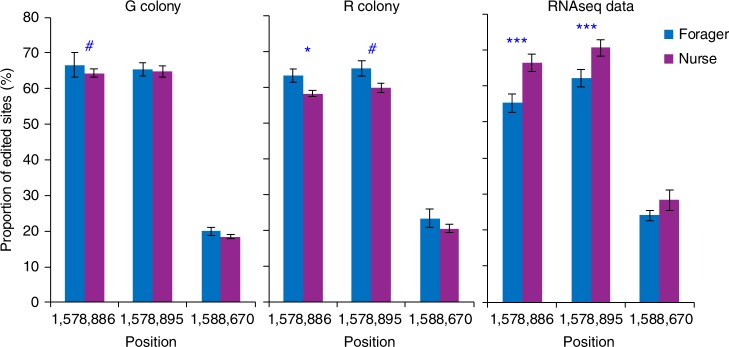
TipE-like transcripts differentially edited in nurse and forager bumblebees. The left and median panels show quantifications of Sanger sequencing for bees from two different field foraging colonies (G and R, respectively). The rightmost panel summarizes the results for the same sites based on MuTect analyses from bees from a different field foraging colony. The numbers on the *x*-axis denote the position of the editing site at Group1.8. The blue marking above the bars summarize the statistical analyses (Mann–Whitney test, ^#^*P* = 0.07, *P < 0.05, ***P < 0.001), values presented as mean ± SE. Note that overall editing levels for all three sites were similar for the three analyses. Source data are provided as a Source Data file

Global editing profile seems to correlate with task performance, but the differences at each individual editing site are subtle and hard to pinpoint. We found only thirteen sites (ten recoding and three synonymous) with a small (up to 11%) but statistically significant (*χ*^2^-test, FDR ≤ 0.05) differential task-related edit (Table 3 and Supplementary Data 9). With the exception of one gene, these genes did not differ in their transcript abundance between nurses and foragers (see column AO in Supplementary Data 9). We tried to validate two of these 13 sites by Sanger sequencing. The tipE (also appearing as “C21orf59-like isoform 1”) transcript of bees was sequenced from individuals of two colonies unrelated to the bees analyzed by RNA-seq. Editing levels were overall very similar for the two colonies and consistent with the results of RNA-seq, lending credence to our bioinformatic analyses of the RNA-seq data. However, the subtle difference between foragers and nurses was not reproduced (Fig. 5). It appears that under natural conditions, the regulation of RNA editing, in at least some specific sites, is complex, and may be influenced by genetic and environmental variation that could differ between the colonies used for the RNA-seq and Sanger analyses. We therefore focused on the global level of editing in bees performing foraging or nursing activities.

**Table 3:** Differential task-related editing in protein coding sites

Region	Position	Strand	Chi-square after FDR (≤0.05)	mRNA ID	Gene description	Codon change (AA)	Difference editing level (N– F)
Group8.1	1217040	−	4.0E-12	XM_003397078.1	Hypothetical protein LOC100643575	Syn: GTA (V;Val) ⇒ GTG (V;Val)	7%
Group5.1	6941896	−	4.0E-12	XM_003395620.1	Glucose dehydrogenase [acceptor]-like	AAC (N;Asn) ⇒ GAC (D;Asp)	1%
Group9.1	1129822	+	2.0E-04	XM_003397380.1	Leukocyte receptor cluster member 8 homolog	AGA (R;Arg) ⇒ GGA (G;Gly)	5%
Group1.8	1578886	+	2.6E-04	XM_003393369.1	Uncharacterized protein C21orf59-like isoform 1	ATC (I;Ile) ⇒ GTC (V;Val)	11%
Group9.6	159925	+	1.1E-03	XM_003397944.1	Retinoic acid receptor RXR-alpha-A-like isoform 2	Syn: CTA (L;Leu) ⇒ CTG (L;Leu)	2%
GroupUn981	1397928	−	2.8E-03	XM_003403010.1	Phosphatidylinositol-binding clathrin assembly protein-like	AAG (K;Lys) ⇒ AGG (R;Arg)	−5%
Group1.8	1578895	+	7.3E-03	XM_003393369.1	Uncharacterized protein C21orf59-like isoform 1	AGC (S;Ser) ⇒ GGC (G;Gly)	8%
Group3.4	1216999	−	8.5E-03	XM_003394411.1	Glutamate [NMDA] receptor subunit 1-like	TAC (Y;Tyr) ⇒ TGC (C;Cys)	7%
Group17.2	237069	+	2.0E-02	XM_003402039.1	Sodium channel protein 60E-like	ATG (M;Met) ⇒ GTG (V;Val)	5%
Group1.3	142051	+	2.0E-02	XM_003393100.1	Hypothetical protein LOC100648310	Syn: GTA (V;Val) ⇒ GTG (V;Val)	11%
Group10.1	6425748	+	2.0E-02	XM_003398309.1	Potassium voltage-gated channel protein *Shab*-like	ACG (T;Thr) ⇒ GCG (A;Ala)	9%
Group17.2	239546	+	2.5E-02	XM_003402039.1	Sodium channel protein 60E-like	AAG (K;Lys) ⇒ AGG (R;Arg)	6%
Group10.1	6425707	+	3.9E-02	XM_003398309.1	Potassium voltage-gated channel protein *Shab*-like	TAC (Y;Tyr) ⇒ TGC (C;Cys)	7%

Task-specialization in bumblebees does not follow a sharp dichotomy, and there are no distinct worker castes or behavioral states as in ants and honeybees (respectively). We therefore, defined a task-specialization index as the number of observed nursing events minus the number of observed foraging trips, and correlated this index with the editing levels observed across the 15 bees for each of the 149 editing events in coding sites. These site-specific measured correlation coefficients are inherently noisy, due to the rather small number of bees, the limited observation time, the rather large error in editing level quantifications and the presumed small effect of task-specialization on editing levels in bumblebees. Indeed, we observe a rather wide distribution of correlation coefficients, none of which is individually significant. Nevertheless, under the null hypothesis of editing activity independent of task performance, one would expect the observed coefficients to be symmetrically distributed around zero, with a similar number of positive and negative values. In contrast, we found that the majority of correlation coefficients are positive; 68.5% [102/149] of the sites in coding sequences, and 68.1% [1779/2611] of all well-covered sites (*P* = 1e-5 and *P* = 1e-75, respectively; two sided proportion test). Accordingly, the distribution is significantly shifted towards positive values (*P*-value = 2.6e-11, *t*-test; Fig. 4b, c). Thus, whereas the effect for each individual site is too small to be confidentially identified with the available sample size, the global behavior of the correlation coefficients demonstrates a significant tendency towards higher editing level in bees performing more nursing than foraging activities. Consistent with this premise, we find a weak but statistically significant positive correlation between the editing level (averaged over the 149 coding sites, see Methods) and the task-specialization index (Fig. 4d, e). Taken together, these analyses support a possible association between task activity (nursing or foraging) and the RNA editing pattern.

In the two other experiments, we did not find differential editing for bees differing in dominance (dominant compared with subordinate in small queenless groups; Experiment 2) or for which we manipulated JH levels (Exp. 3, data not shown).

### Regulation of BtADAR expression by the social environment

**Fig. 6:**
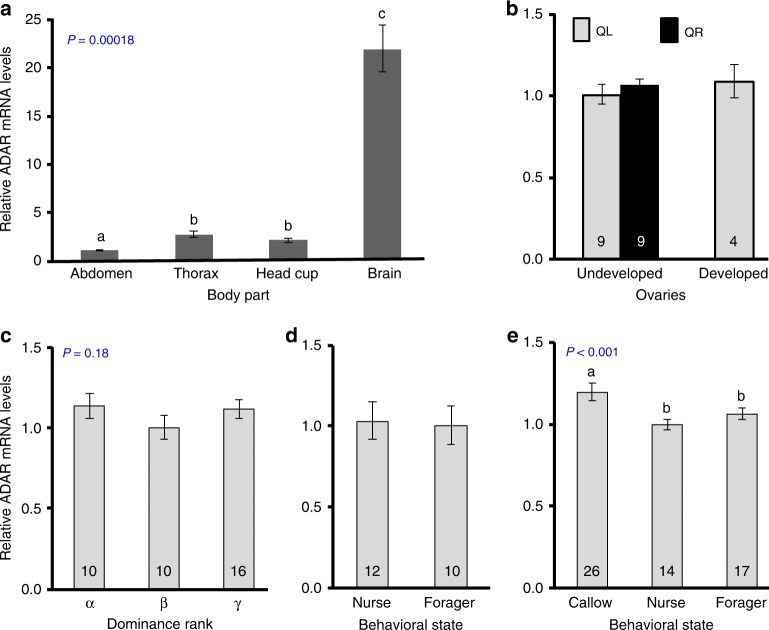
ADAR transcript abundance. **a** BtADAR mRNA levels are enriched in the brain, compared with the rest of the body. The *P*-value was obtained from a Kruskal–Wallis analysis; body parts marked with different letters differ in a Mann–Whitney test with Bonferroni correction for multiple comparisons. Sample size was six bees. **b** Ovarian state and queen presence do not influence brain BtADAR mRNA abundance (Two-way ANOVA; ovarian development, *P* = 0.42; queen presence, *P* = 0.41); QR = queenright colonies, QL = queenless (orphan) groups of bees). **c** Dominance rank in groups of three queenless workers did not affect brain BtADAR mRNA levels (One-way ANOVA test, *P* = 0.18). **d**, **e** Nurses and foragers have similar brain ADAR transcript levels in free-foraging queenright colonies of *B. terrestris* (**d**, Mann–Whitney test *P* = 0.39) and honeybees (**e**, one-way ANOVA test, bars with different small letters differ in a Least Significant Difference (LSD) post hoc test, *P* < 0.001). Values presented as mean ± SE. Sample size is shown inside each column (besides A). Source data are provided as a Source Data file

BtADAR is significantly enriched in the brain (Kruskal–Wallis test: *χ*^2^ = 19.867, df = 3, *p* = 0.00018; Fig. 6a), where BtADAR abundance is about 20-fold higher than in the abdomen. Given that one of the most obvious factors that may regulate editing levels is differential expression of the ADAR enzyme we further compared brain BtADAR abundance in bees differing in behavior or reproductive state. We found no differences between dominant and subordinate bees in our RNA sequencing dataset (FDR adjusted *P*-value = 0.86, data not shown), or in an independent experiment in which we also compared bees developing in queenless or queenright colonies (Fig. 6b, c). Juvenile Hormone, which regulates ovarian activity and influences dominance, does not affect BtADAR transcript abundance (adjusted *P*-value = 0.49, data not shown). In addition, brain BtADAR expression did not differ between foragers and nurses in the samples used for the RNA editing analyses (adjusted *P*-value = 0.83, data not shown), as well as in an independent experiment with bees from different source colonies (Mann–Whitney test *P* = 0.39; Fig. 6d). Our analyses, however, suggest a very weak effect of age: BtADAR expressions seems to increase with age (multiple regression for pooled analyses of QL and QR bees; *R*^2^ = 0.11, *P* = 0.006, no influence of queen presence: *P* = 0.74). Consistent with this analysis, there is a significant difference between age groups (Two-way ANOVA, age: *F* = 3.17, *P* = 0.02, queen presence: *F* = 0.074, *P* = 0.78; the difference between 3 and 6 days of age were statistically significant in a Bonferroni post hoc test; *P* = 0.001; Supplementary Fig. 6).

We further tested the relationships between task performance and brain ADAR expression in the honeybee *A. mellifera*, in which the division of labor relates to age and is associated with more stable behavioral states compared with bumblebees. We found that ADAR levels in newly emerged bees are slightly (~20%) but statistically significantly higher than in both normal age (7-day-old) nurses and normal age (21-day-old) foragers (one-way ANOVA with LSD post hoc test, *P* < 0.001; Fig. 6e). As in the bumblebee, brain amADAR expression was similar for nurse and forager honeybees.

## Discussion

We studied A-to-I RNA editing in naturally behaving animals in an ecologically relevant setting. We found extensive RNA editing in 1.15 million unique genomic sites, mostly within non-coding regions. The extent of hyper-editing in the bumblebee, higher than that reported for brains of humans and other mammals (Fig. 1c; ref. ^33^), is surprising considering that we show that bees, like other insects, have lost ADAR1, and express only a single ADAR enzyme^11^. It is generally accepted^42–44^ that the primary role of the widely expressed ADAR1 in vertebrates is to edit endogenous long dsRNAs, in order to prevent an innate immune response triggered by such endogenous transcripts. On the other hand, mammalian ADAR2 is expressed primarily in the brain and arteries^33,45^, with a primary role of targeting isolated editing sites within coding sequence. Consistent with this view, Drosophila species that express a single ADAR enzyme homologous to ADAR2, exhibit a relatively low level of hyper-editing (Fig. 1c; ref. ^33^), but do show hundreds of recoding sites. Our transcript abundance analyses show that BtADAR expression is enriched in brain tissue. Thus, the comparison of our findings for the bumblebee with other species challenges the common view by providing an example of extensive editing of non-coding sequences in an insect lacking an ADAR1 homolog. These findings suggest that the bumblebee ADAR enzyme (and probably that of other insects) can take on some of the functions performed by ADAR1 in mammals, and partially compensate for the lack of a second editing enzyme. Two-hundred and nineteen of the editing sites reside in protein coding sequences, 164 of which are recoding sites that can potentially modify protein function. Remarkably, some of these sites are similarly edited in other insects and even in cephalopods and mammals. Given these prevailing nonsysnonymous substitutions (recoding) we further compared brain editing and ADAR transcript abundance in bees differing in their behavior, physiology, or social environment.

Recoding by RNA editing is a relatively subtle regulation mechanism, enabling dynamic fine-tuning of the transcriptome and proteome in response to changing external, internal, or developmental conditions^24,46^. Social insects are specifically interesting in this context because they show substantial behavioral, physiological and morphological plasticity that is largely regulated by their social environment. In many species, including bumblebees, considerable phenotypic variation is exhibited by genetically similar individuals, suggesting that genetic variation plays a minor role, if any. The only previous study on RNA editing in social insects compared morphologically distinct castes of the highly social leaf-cutting ant *Acromyrmex echinatior*^25^. This study reported a consistent editing pattern for each of the three castes considered (gynes, large workers, small workers), but these sites recoded overall only 34 protein coding genes, only three of these are also edited in other ant species that they tested. The differences between queen and worker ants are considerable, many of which are determined during development, and many are fixed for life. Here, we asked whether RNA editing can be associated with relatively short-term changes during the adult stage of bumblebee workers. We found that brain RNA editing was not influenced by dominance rank (which is associated with aggressiveness) or JH (which regulates fertility and aggression in bumblebees) manipulation, but was correlated with the task the worker bee performs. Similarly to the ants, while the global editing pattern could be correlated with behavior, very few individual sites could be pinpointed as being modified. Admittedly, the association between task performance and RNA editing in the brain is rather weak. However, task-specialization in bumblebees is also relatively weak and flexible, with many workers switching between brood care and foraging activities within the same day. Moreover, we analyzed the whole brain, which is expected to mask significant task-related differences in RNA editing that are restricted to specific brain regions. The notable enrichment for ion channels and other genes involved in neurotransmission among the RNA edited transcripts lend credence to the hypothesis that A-to-I editing in the bumblebee affect neuronal functions.

We found editing events in many ion channels, including five potassium voltage-gated channel transcripts (*eag*, *Shab*, *Shal*, *Shaw*, and subfamily KQT member 1(XM_003396133.1), calcium-activated potassium channel (slowpoke), voltage-dependent calcium channels, and sodium channels. Some of these ion channels were edited in multiple sites (e.g., *Shab* in 14 sties, *Shal* in 5 sites, *sodium channel protein 60E*-like in 6 sites). This is noteworthy because relatively small changes in the ion channel amino acid sequence, such as those created by non-synonymous RNA editing, can have significant influence on the ion channel permeability or regulation. Unlike mammals, insects typically have only a single paralog for each subunit of a given ion channel gene^47,48^, and functional diversity is achieved by means of alternative splicing and RNA editing that generate variants differing in their physiology and pharmacological properties^48,49^. Indeed, transgenic manipulation analyses in model organisms have shown that editing in orthologs of the ion channels that are edited in the bumblebee affect the channel biophysical and physiological properties, which in turn can alter behavior^50–54^. This evidence lends credence to the premise that RNA editing in bumblebee is also functionally significant. We further focus on *Shab* because there is much detail on the functional outcome of editing its transcript in similar voltage-activated potassium K_v_ channels^53,54^. Additionally, we found 14 different edited sites in the *B. terrestris Shab* protein, two of which were differentially edited between worker bees performing nursing and foraging activities.

The extensive editing in *BtShab* is similar to that observed in cephalopods and significantly higher than in mammals K_v_ channels. Voltage-activated potassium channels constitute four identical subunits (homotetramer), each with six transmembrane domains. The first four transmembrane domains form the voltage-sensing domain, while the last two form the pore domain (Fig. 2). Depolarization lead to conformational changes within the voltage sensor driving the opening of an activation gate located at the intracellular end of the permeation pathway. Four of the identified editing sites are mapped to the pore forming 5th and 6th transmembrane domains and are conserved in *Drosophila*, three of which are also conserved in other insects^38^ and cepalophods (Table 2). Functional studies have shown that editing of these sites modified the rate of channel closing and inactivation^53,54^. Given the conservation of these editing sites (Table 2 and Fig. 2), it is very likely that the extensive editing in these sites in bumblebee *Shab* has similar functional consequences. It is notable that eight of the editing sites, including the two that differ between nurses and foragers are mapped to the intracellular N-terminal domain, which appears to be less conserved. The functional significance of recoding in this part of the ion channel is currently unknown.

What can account for the observed differences in editing between bees differing in the degree of brood care and foraging activity? In terms of the underlying molecular mechanisms, this correlation was not associated with variation in brain BtADAR transcript levels (Fig. 6d). This finding is consistent with other studies showing that ADAR RNA or protein levels do not fully account for the spatiotemporal changes of RNA editing^55^. Autoediting that was shown to regulate ADAR editing activity in mice and flies, did not differ among bees differing in the nursing/foraging activity ratio. Thus, other mechanisms seem to up-regulate A-to-I editing activity in nurses. Foraging bees experience a more variable environment in which factors such as temperature and humidity can vary substantially, and may experience stress both in terms of physical endurance and predation risk. The environment of nursing bees is different; they remain in the safe, dark, and relatively stable environment of the nest. Foragers also experience extremely rich visual environments, and their olfactory and auditory world is different than inside the nest. The observed differences in RNA editing may also be related to the internal neuroendocrine or neural processes underpinning behaviors associated with brood care or foraging activity. Given that body size is positively correlated with the propensity to perform foraging activities^56^, it is also possible that developmental processes that determine body size^28^ contribute to the observed task-related variation in RNA editing.

In summary, we performed the first brain editome analyses of a bee, and the first in which RNA editing was studied in the context of natural behavior in an ecological context. Our analyses show that brain RNA editing in the bumblebee is extensive and includes recoding of many ion channels and other proteins regulating important neuronal functions. Overall RNA editing, as well as protein recoding, correlate with the task the bee performs. These findings suggest that the modulation of RNA editing may include relatively short-term processes associated with foraging or brood care activity and may not be limited to relatively longer-term processes such as temperature acclimation as is currently commonly assumed.

## Methods

### Bees

*Bombus terrestris* colonies for the BtADAR expression analyses were purchased from Pollination Services (Yad-Mordechai, Israel). *Incipient colonies* contained a queen, approximately ten to fifteen workers and brood at all stages of development. “*Mini-Colonies*” were obtained about 2 weeks after first worker emergence and contained a queen, ~20 workers and brood at all stages of development. Each colony was housed in wooden observation boxes (30 × 23 × 20cm) with a transparent Plexiglas cover. All the colonies were placed in environmental chambers (29 ± 1 °C, 50 ± 5 relative humidity (RH)) at the Bee Research Facility at the Edmond J. Safra campus of the Hebrew University of Jerusalem, Givat Ram, Jerusalem, Israel. Bees were fed ad libitum with commercial sugar syrup and a processed pollen (fresh pollen mixed with sugar syrup), all of which were purchased from Pollination Services (Yad-Mordechai, Israel). All observations and treatments were conducted under dim red light, to which bees are not visually sensitive. As an index for body size, we measured the length of the Marginal cell in the front wings^28^. Bees collected for molecular analyses were flash-frozen in liquid nitrogen (−196 °C), immediately transferred to dry ice, and then stored in an ultra-low freezer (−75 °C) until further analysis.

For the RNA sequencing experiments, we purchased incipient colonies (5–10 workers and brood at all stages of development) from BioBee Biological System (Sde Eliyahu, Israel). Each incipient colony was placed in a wooden nesting box (21 × 21 × 12 cm) with a front wall and cover made of transparent acrylic plastic (Plexiglas^TM^). We placed the nesting boxes with the bees in an environmental chamber (28 ± 1 °C; 50 ± 5% RH**)** under constant darkness, in the Bee Research Facility, and fed them as described above. For the RNA-seq experiments we used workers from 12 colonies; eight for the juvenile hormone and dominance rank experiments; and four for the worker division of labor experiment. In order to minimize genetic variation among treatments, all colonies used for these experiments were headed by full-sister queens that are genetically very similar (all have the same paternal chromosome; *r* = 0.75). These full-sister queens were crossed with unrelated drones and induced to found colonies according to standard industry protocols.

Honeybees (*Apis mellifera*) were collected from freely foraging colonies maintained according to standard beekeeping techniques at our Bee Research Facility (for details see ref. ^57^). The honeybees were derived from a mixture of European races (mostly Italian), which are typical to Israel.

### The ADAR orthologs of *Bombus terrestris* and *Apis mellifera*

We used tBLASTX to search for ADAR genes in the genomes of *Bombus terrestris* (version Bter_1.0) and *Apis mellifera* (version Amel_4.5). Details of the primers and amplicons of these transcripts are summarized in Supplementary Table 1.

### Tissue dissection and RNA extraction

We first separated the heads and bodies on dry ice, and then opened the head capsule cuticle and partially lyophilized the head for 60 min in SpeedVac (Heto DryWinner) to facilitate the dissection of the brain. Following freeze-drying, we opened the head capsule and removed the brains. We performed the dissection on deeply frozen aluminum stage immersed in dry ice such that the tissue remained frozen during the entire procedure. We extracted total brain RNA using the Invisorb Spin Tissue RNA Mini Kit (Invitek GmbH, Berlin, Germany). The RNA was stored in an ultra-low freezer until shipped on dry ice for sequencing at the Carver Biotechnology Center at the University of Illinois at Urbana-Champaign, IL (UIUC).

### RNA and DNA sequencing

RNA quality was assessed at UIUC with Agilent Bioanalyzer (Agilent Technologies, Santa Clara, CA). We prepared RNA-seq libraries with unique barcodes using Illumina’s TruSeq RNA-seq Sample Prep kit. Libraries were quantified by qPCR and pooled before sequencing. We sequenced the pooled libraries on eight lanes for 101 cycles from each end using a TruSeq SBS sequencing kit (version 3) on an Illumina HiSeq2500. We used Casava 1.8.2. to generate Fastq files.

For the DNA sequencing we extracted DNA from leg and wing tissues of four bees using the MOBIO power soil DNA extraction kit (MOBIO Laboratories (now Qiagen), Carlsbad, CA). Shotgun genomic libraries were prepared using the Kapa Hyper-Prep kit (Kapa Biosystems Inc., Wilmington, MA). Libraries were quantified by qPCR and sequenced for 161 cycles from each end on an Illumina HiSeq2500 using TruSeq sequencing kit version 1. We generated and demultiplexed Fastq files with the bcl2fastq v1.8.4 Conversion Software (Illumina, San Diego, CA).

### qPCR expression analysis

Total RNA was isolated with the Bioline Isolate II RNA Mini Kit (cat# 52073) according to the manufacturer’s instructions. Two-hundred nanograms of total RNA was reverse transcribed with (Bioline cat# 27036) to obtain cDNA template. To measure RNA levels, we used real-time quantitative RT-PCR (qPCR) with an ABI’s STEP-ONE Real-Time PCR sequence analyzer using a fast SYBR green detection protocol (ThermoFisher cat# 4385612). For each biological sample, we measured three technical replicates. For brain RNA analyses we used *Elongation Factor 1a (EF1a)* as the housekeeping control gene as it was previously shown not to vary between the brains of bees showing different behaviors (e.g., refs. ^58,59^). For experiments comparing mRNA levels across different tissues we normalized ADAR mRNA levels relative to two control genes, RPL13 and S16, selected based on a series of preliminary studies (see Supplementary Methods and Supplementary Table 2). For the graphical display of our qPCR analyses we used the ∆∆Ct method adjusting the sample with the lowest expression level to a value of 1.0. All the statistical tests were performed on ΔCT values that were normally distributed.

### Determining BtADAR mRNA levels in different body parts

We housed two incipient colonies in nest boxes that we placed in an environmental chamber. Colonies were connected to the outside with a transparent plastic tube allowing worker bees to forage for nectar and pollen outside; we supplemented the colonies with pollen or nectar as necessary. One week after connection to the outside, when the colonies were self-supported and before the Competition Point (CPh, determined as in ref. ^60^), six newly emerged bees were collected (easily identified based on the lack of yellow pigmentation). We dissected the collected bees on dry ice to obtain the following body parts: head capsule (without the brain), brain, thorax, and abdomen.

### Influences of social behavior on brain ADAR mRNA levels

We performed a series of experiments to test whether brain ADAR transcript abundance is influenced by the social environment or behavior of worker bees. In the first experiment we tested the influence of the queen presence and dominance rank on brain BtADAR mRNA levels. We used four “mini” colonies; two of which were marked for observational purposes, and two were used as “*donor colonies*”, providing newly emerged callow workers. The newly emerged workers were each marked with an individual number tag (Graze, Germany, Cat# 1374), and randomly distributed to queenless (QL) or queenright (QR) treatments. QR bees developed in a colony with a queen, workers (up to 30), and brood at all stages of development; QL workers developed in triplets without a queen. Each QL group was housed in a small wooden cage with transparent walls (10 × 11 × 5 cm). We placed the QR colonies and the QL groups in the same environmental chamber with pollen and nectar ad libitum. We used bees of similar body size within each cage in order to avoid the influence of body size on dominance hierarchy^61^. Tagging and assignment of bees to treatments occurred over a 1-week period. We collected all the tagged bees 2 weeks after the first tagging day. Thus, the bees in this experiment were 6–12 days of age. Dominance rank was determined as in previous studies (e.g., ref. ^62^; Experiment 2 in the Supplementary Methods). The most dominant bee in each QL group was designated as the ‘α bee’, the second ranked ‘β’, and the most subordinate ‘γ’. We compared QL and QR bees, as well as QL bees differing in the dominance rank. Given that dominance behavior is correlated with ovarian state (e.g., refs. ^61,62^), we also compared worker bees with developed and undeveloped ovaries. We used the length of the largest terminal oocyte as an index for ovarian state.

In the second experiment, we tested the influence of task performance on brain BtADAR mRNA levels. We used two incipient colonies that were connected to the outside. Four additional incipient colonies were used as sources for newly emerging bees (“donor colonies”). Starting 1 week from connection to the outside and throughout the following week, newly emerged callow bees were collected from the experimental (focal) and donor colonies. Each collected bee was marked with a colored plastic number tag (Graze, Germany, Cat# 1374) and immediately introduced randomly into one of the experimental colonies. Two days after tagging we started sessions of behavioral observations. Foraging observations were performed at peak activity times of the day, i.e., 4 h in the morning (06:00 to 10:00 a.m.), and two in the afternoon (4:00 to 6:00 p.m.). Foraging activity was recorded when a bee left the colony in a direct line of flight. This flight pattern is distinct from ‘orientation flights’ that are characterized by circular maneuvers in front of the hive entrance. We recorded the number tags of foraging bees returning to the colony with conspicuously visible pollen loads on their hind legs. We performed additional observations inside the nest box during a session between 11:00 a.m. and 1:00 p.m. in which we recorded brood-feeding behavior as a proxy for nursing behavior. Observations and collections of bees for RNA analyses were carried out when the colonies were already self-supported and before the ‘*Competition Point*’ (refs. ^60,63^). All bees were collected on a single day, when they were 4–9 days of age. For the analyses of task influence on ADAR mRNA levels we included only bees which showed either foraging or nursing, but not both.

For comparison we also tested the influences of age and task performance on brain AmADAR levels in honeybees, in which the division of labor relates to age. We used two honeybees (*Apis mellifera*) colonies that were maintained using standard beekeeping procedures in our Bee Research Facility. In order to obtain newly emerged worker honeybees, we removed three honeycomb frames with sealed brood, each from a different field source colony. We brushed all the adult bees, placed each frame in a wooden box, and transferred it to an incubator (32 ± 1 °C and 55 ± 5% humidity). Bees emerging over the night were color marked (a distinct color for each source colony), and reintroduced back into their source colony. Given that task activity in honeybees relates to age, we repeated this procedure two times: first tagging bees for foraging observations, and 2 weeks later, for nursing observations. Three weeks from the first tagging day, and 1 week following the second tagging, we collected the tagged bees. Three-week-old bees captured when returning to the hive with pollen were classified as ‘foragers’; 7-day-old bees inserting their heads into brood cells were classified as ‘nurses’. On the same day, we also collected newly emerged bees.

### Identification of hyper-edited reads and sites

Putative hyper-editing sites were identified as described in Porath et al.^30^, with default parameters. This method focuses on clusters of edited sites that are usually overlooked by standard alignment methods. Briefly, as edited RNAs show guanosines (G) in sites with genomic adenosines (A), RNA sequences with multiple editing sites are widely different from their corresponding DNA. The computational screen overcomes this obstacle by masking potential editing sites in unaligned reads^30^. As an input we used 58 RNA-seq datasets (paired-end 100 bp reads) and the *Bombus terrestris* genome assembly version Bter1.0. As the *B. terrestris* genome draft is still rather fragmented two paired-end reads may be mapped to different scaffolds, alignment that would be considered improper. We thus treated each paired-end read as independent single-end reads. We searched for high-quality sequencing reads (Phred score ≥ 30) containing at least five A-to-G mismatches per 100 bp sequence, and for which the A-to-G mismatches comprise > 60% of the total number of DNA–RNA mismatches. To validate the specificity of the pipeline, we used the same procedure to search for all other possible types of mismatches (A-to-C, G-to-A, and so on). We could not distinguish between a given mismatch and its complementary one, since the RNA-seq reads could be either sense or antisense (non-stranded library). Thus, although there are 12 possible single-nucleotide mismatches, we report results for only six categories of editing events: A-to-G, G-to-A, A-to-T, A-to-C, G-to-C, and C-to-A and each category represents both the forward and complementary mismatch. For genes and coding region annotations we downloaded the NCBI RefSeq annotations from the BeeBase site (http://hymenopteragenome.org/beebase) on 7 May, 2014.

### Defining hyper-edited clusters, regions and dsRNA structure

Hyper-edited clusters were defined as the genomic sequence between the first to the last A-to-G mismatch in each hyper-edited read^30^. Overlapping clusters were merged to create a hyper-edited region, maintaining a maximum distance of 20 bp between clusters. The main targets of ADAR enzymes are long dsRNA structures^64^. To test whether the hyper-edited regions occur within such structures, we aligned the DNA sequence of each hyper-edited region (found in one representative sample) to the genomic sequence spanning 2 kbp upstream and 2 kbp downstream of the region, looking for reverse-complement matches that may indicate a possible dsRNA structure. As a control, we looked for same-strand matches (other than the trivial one). As an additional control, we repeated the search for random regions of similar lengths. For these alignments we used bl2seq^65^ with parameters -F F -W 7-r 2, keeping only alignments with at least 65% complementarity along 60% of the hyper-edited region length.

### Computing editing levels

To estimate editing levels for each hyper-edited site, we first aligned the RNA-seq datasets to the *B. terrestris* genome with the STAR 2-pass aligner, using the default parameters^66^. We then used Picard (http://broadinstitute.github.io/picard/) and GATK^67^ software packages with the following tools ‘picard MarkDuplicates’, ‘picard ReorderSam’, ‘GATK SplitNCigarReads’, ‘GATK RealignerTargetCreator’, ‘GATK IndelRealigner’, and ‘Picard FixMateInformation’. We further used REDItools^68^ with parameters: -v 1 -n 0.01 -c 1 -T 6–6 to estimate the level of A-to-I editing. Editing index over sites was measured as the total number of Gs divided by the total number of Gs + As^69^.

### Detecting RNA editing using DNA-seq and RNA-seq datasets

In a separate set of analyses, we detected putative RNA editing sites by comparing DNA and RNA sequences. To get a DNA genomic reference for our bumblebee population, we sequenced the genome of four representative bees (out of the 58 for which we sequenced the brain transcriptome). We used BWA^70^ with default parameters and STAR 2-pass^66^ to align the DNA-seq, and the RNA-seq to the published *B. terrestris* genome. Alignments were improved by picard (http://broadinstitute.github.io/picard/) and GATK^67^ tools. Detection of putative editing sites was done by MuTect version 1.1.4 (using default parameters), an algorithm that was developed for identifying somatic mutations in cancer samples by comparing the normal genomic DNA sequence with the matched tumor DNA sequence of the same individual^34^. As the “normal” genomic reference sequence we used the consensus sequence generated from aligning the four DNA-seq datasets that we generated. The RNA sequence of each bee was entered in the “tumor sample” option of the MuTect program. We have also attempted detection by the well-used REDItools DNA–RNA tool^68^, with different combinations of parameters. However, the resulting A-to-G fraction for all genomic sites was only 40–43%, substantially lower than the 64% obtained by MuTect.

### Detecting conserved recoding editing sites

To detect recoding sites conserved between bumblebees and *Drosophila* flies, we aligned the bee protein sequences harboring recoding sites to the sequences of the proteins harboring recoding sites previously identified as conserved across *Drosophila* species^35^. We identified seven sites conserved between flies and bees, where recoding occurs at the homologous amino acid in both species, resulting in the same non-synonymous alteration. Editing levels of those conserved sites in the fly were computed using data from Duan et al.^36^.

### The influence of behavior and physiology on brain RNA editing

We performed three experiments testing the influence of behavior or physiological state on brain RNA editing. In Experiment 1 we tested the effect of task performance focusing on workers differing in the levels of brood care (“nursing”) and foraging activities. We placed four colonies in an environmental control room (28 ± 1 °C; 40 ± 5% RH). Two of the colonies were connected to the outside with a 1 m long clear plastic tube, and the remaining two were used as donor colonies. We collected newly emerged bees from the free-foraging colonies, tagged them with individual number tags (as above), and returned them to their mother colony for 2 days. We similarly collected and tagged bees from the two donor colonies, and introduced them randomly into the two foraging colonies. In order to avoid increasing the total numbers of bees in the colonies, which can affect colony development^46^, one bee was removed for each two bees introduced^60^. We marked and introduced a total of 35 bees into each free-foraging colony over 2 successive days. When the tagged bees were 3–4 days of age, we started behavioral observations that continued over 2 days. We observed the colonies for 3 h in the morning (07:00–10:00 a.m.), and 2 h during late afternoon (4:00–6:00 p.m., before sunset). We focused on foraging and brood care activities because these behaviors are ubiquitous, well characterized, crucial for the division of labor and colony performance, and easy to identify. Each observation session was divided between 1 h in which we recorded foraging activity next to the nest entrance, and 1 h in which we recorded larval feeding behavior inside the room. Larval feeding behavior was composed of a sequence of acts starting with the worker bee opening a brood cell, inserting her mouthparts into the open cell, and regurgitating food. Food regurgitation is characterized by the typical contraction of the abdomen and takes a few seconds^56^. RNA was sequenced from four foragers and four nurses from each colony. The foragers were defined as bees that were observed conducting at least three foraging trips and were not observed feeding larvae (with the exception of one forager that performed two feeding behaviors and eight foraging trips). Nurses were defined as bees observed performing at least three feeding acts, and were not seen foraging at all. We flash freezed the focal bees in liquid nitrogen following the second day of observations when they were 5 to 6 days of age. We transferred the frozen bees to marked tubes immersed in dry ice and stored them in an ultra-low freezer until further analysis.

In Experiment 2 we compared queenless (i.e., orphan) workers differing in dominance rank (dominant vs. subordinate). In Experiment 3 we compared bees with experimentally manipulated JH levels. The details of Experiments 2 and 3 are summarized in the Supplementary Methods. For each individual RNA-seq dataset, editing levels in 219 sites located within protein coding sequences were quantified and compared. We limited the analysis to sites with a median coverage of at least 50 reads per sample. We used *χ*^2^-tests with Benjamini–Hochberg multiple-testing correction, setting the false discovery rate to 5%.

### Reporting summary

Further information on experimental design is available in the Nature Research Reporting Summary linked to this article.

## Supplementary information

Supplementary Information

Peer Review File

Reporting Summary

Description of Additional Supplementary Files

Supplementary Data 1

Supplementary Data 2

Supplementary Data 3

Supplementary Data 4

Supplementary Data 5

Supplementary Data 6

Supplementary Data 7

Supplementary Data 8

Supplementary Data 9

Source Data

## Data Availability

A reporting summary for this Article is available as a Supplementary Information file. The accession code for all the sequencing data that was used in the article is PRJNA497863. All other relevant data are provided in the Supplementary Information files or available from the corresponding authors on request. The source data underlying Figs. 1a–e, 3, 4a–e, 5 and 6 and Supplementary Figs. 2, 3, 4 and 6 are provided as a Source Data file.
