# Supplementary material for: RNA editing is abundant and correlates with task performance in a social bumblebee
Source: Nat Commun. 2019 Apr 8;10:1605. doi: 10.1038/s41467-019-09543-w (PMC6453909; doi:10.1038/s41467-019-09543-w)
Supplement: Supplementary file 1 — Supplementary Information [file 41467_2019_9543_MOESM1_ESM.pdf]

Supplementary information

RNA editing is abundant and correlates with task performance in a social bumblebee

Hagit T. Porath[1]*, Esther Hazan[2]*, Hagai Shpigler[2], Mira Cohen[2], Mark Band[3], Yehuda Ben Shahar[4], Erez Y. Levanon[1], Eli Eisenberg*[5], Guy Bloch*[2]

1) The Mina and Everard Goodman Faculty of Life Sciences, Bar Ilan University, Ramat-Gan, Israel

2) Department of Ecology, Evolution & Behavior, Alexander Silberman Institute of Life Sciences, Hebrew University of Jerusalem

3) Roy J Carver Biotechnology Center, The University of Illinois at Urbana-Champaign, IL, USA, and Institute of Evolution, University of Haifa, Israel

4) Department of Biology, Washington University in St. Louis, St. Louis, MO

5) Raymond and Beverly Sackler School of Physics and Astronomy and Sagol School of Neuroscience, Tel Aviv University, Tel Aviv, Israel

* - These authors contributed equally

# - corresponding authors

Emails of corresponding authors:
Guy Bloch: guy.bloch@mail.huji.ac.il,
Eli Eisenberg: elieis@post.tau.ac.il.

Supplementary Tables

Supplementary Table 1. Primers used for qPCR amplification of ADAR transcripts in *B. terrestris* and *A. mellifera.*

Species	Forward primer	Reverse primer	Tm Cº	Amplicon length	CDS Location	E%
A. mellifera	TGCTTATGGGCC AAAGACTG	ACTGCCAAGAAC GATGCTGT	59.2	134bp	V1&V2,<br>F: 1400-1419<br>R: 1513-1533	96.3
B. terrestris	GAGCAAGAGGTC AGTTGCGAACC	CGACATCGTCAG CAGTCTTTGGC	60.1	122bp	V1, F: 1310-1329<br>R: 1409-1431<br>V2, F: 1274-1293<br>R: 1373-1395	98

Supplementary Table 2: Selection of normalization control genes for experiments comparing mRNA levels among different body parts of *Bombus terrestris* workers. **A**. Analysis summary for the geNorm program. The M values represent the variance. **B.** Analysis summary for the NormFinder program. The stability values represent the variance. **C.** Analysis summary for BestKeeper program: r represents coefficient of correlation values.

		**Ef-Tu**	**RPL13**	**S16**
**A.**	M-Value	0.987	0.4997	0.4997
**B.**	Stability value	0.040	0.030	0.017
**C.**	Coeff. of corr. [r]	0.153	0.967	0.879
	P-value	0.205	0.001	0.001

Supplementary Methods

Primers used for qPCR amplification of ADAR transcripts in B. terrestris and A. mellifera

The primer sequences and details are summarized in Supplementary Table 1.

Selection of reference genes for qPCR analyses in different tissues of B. terrestris

We tested several control genes for experiments comparing mRNA levels among different *B. terrestris* tissues. We compared genes that were used as control genes in previous qPCR studies with bumblebees [1] that show little variation in an RNAseq study comparing brain expression in bees differing in task performance, JH levels, or dominance (Shpigler *et al*, unpublished). The candidate genes compared were: *Ribosomal protein L13 variant 1 (RPL13)*, *Phospholipase A2 (PLA2) ,Arginine kinase (Argk), 40S Ribosomal protein S16-like (S16)*, *Elongation factor Tu* (*Ef-Tu*), and *Elongation factor 1a (EF1a)*. Primers were tested on a standard curve using a dilution series of cDNA. The Correlation coefficients (R^2) and the amplification efficacy (%E) were determined based on the standard curve. The amplification efficacy was determined based on the formula: $\mathrm{E}(\%) = 100 * (1 - 10^{-\frac{1}{\mathrm{Slope}}})$. Candidate genes that showed amplification efficacy values below 95% or above 105% were excluded from further analyses. Second, we determined mRNA levels in various tissues of 1-day-old bees. Candidate reference genes showing an expression difference of more than 2 cycles among body parts were excluded from further analysis. Third, we tested the expression variance using 3 designated software programs: geNorm [2], NormFinder [3] and BestKeeper [4]. Each program received the qRT-PCR results and calculated the variance between and within tissues based on different algorithms. All three programs identified *S16* as the most stable (Supplementary Table 2). *RPL13* and *S16* were selected based on the lowest tvariance across the three programs. The primvers used for RPL13 are (the asterisk mark exon-exon boundary): F - GTTGCACCAAG*ACCTGTGAAG; R – AGAAGAACTGAAGGCTAGTGGT. The primvers used for S16 are: F-CACGCACCAGGAAAGGGTAT; R - CCGTCTCAAATTG*GTGTGATTCT. The primers used for Ef1α are: F – CGTTTACCGCTTCAGGACGT, R- GCATGCCTGGTTTCAGAATACC.

Experiment 2. The influence of dominance rank brain RNA editing

We determined the dominance status for individually tagged bees from the control groups in Experiment 3. Dominance rank was determined based on two sets of behavioral observations performed when the bees were three and five days of age. Each observation lasted 20 min. Dominance index was calculated following the method described in Bloch et al. (1996). Briefly, we recorded which bee advanced and which retreated in each dyadic encounter. The dominance index was defined as: (1 – retreats) / total encounters, and ranges between 0 - 1. We sorted the bees based on their dominance index to determine a dominance rank within each queenless group; the individual with the highest dominance index was dubbed "α", the second highest "β", and the one with the lowest value "γ". For RNAseq analysis we used only the α and γ bees.

Experiment 3. The influence of juvenile hormone on brain RNA editing

We manipulated JH levels by allatectomy and treatment with JH-III. We used newly emerged (< 18 hrs post pupal emergence) worker bees. At this age the cuticle of adult bumble bees is relatively soft and easy to manipulate. The collected bees had free access to sugar syrup and pollen *ad libitum* both before and after the allatectomy operation. For the allatectomy treatment (*'CA-'*), we anesthetized the bees on ice for 5-20 min (the variation in chilling duration was due to individual differences in body size, and consequence of the dissections order as the bees were chilled in groups of four). The anesthetized bees were fixed under a stereoscopic microscope (Nikon SMZ645, X50) to an ice-chilled metal stage. The bees were fixed with molded modeling clay such that their dorsal side faces up, and the head bent down to expose the thin neck cuticle connecting the thorax and the head. We used fine scalpel to open a latitudinal incision in the posterior part of the head capsule, and moved the inner membrane and trachea to expose the corpora allata (CA) glands. Both corpus allatum were gently grasped with fine forceps and detached. The entire procedure took between 2-5 minutes. The cuticle resumed to its original shape and the incision appeared self-sealed within few hours after the operation. Sham-operated bees (*'Sham'*) were handled and dissected in a similar way to the CA- bees, but their CA glands were only touched gently and not detached. Control bees (*'Control'*) were anesthetized and handled similarly, but were not operated. At the end of the operation the bees were placed in a small wooden cage (12x5x8 cm), with other similarly manipulated bees, and were left to recover overnight in an incubator (32ºC ± 1ºC, 70%±5% RH). On the second day, the surviving bees from each treatment group were assigned to groups of three, each transferred to a fresh wooden cage (12x5x8 cm). The bees in each group were colored marked for individual recognition and were kept in the incubator (32ºC ± 1ºC, 70%±5% RH) for four days with ad-libitum food supply, 70% sugar syrup and fresh pollen cake. For replacement therapy (*'CA-+JH'*) half of the allatectomized groups were used. The bees were chilled on ice for 3-5 minutes, and when anesthetized treated topically with 70ug of JH-III (Sigma, cat #: J-2000) dissolved in 3.5µl Dimethylformamide (DMF, J.T Backer, cat #: 7032), giving a final concentration of 20 ug/µl. The JH solution was applied to the dorsal part of the thorax. The bees were treated with the JH-III solution twice; the first on day 2 and the second on day 4 (following the protocol developed by Shpigler et al. 2014). Sham and CA- bees were similarly handled and chilled, but only treated with the vehicle (3.5µl DMF). The control bees were chilled, but otherwise untreated. Following treatment the bees were marked individually with dot of color on the thorax (Enamel) and placed back into their original cages. The treatment didn't affect the survival of the bees as the CA-+JH group survival was similar to the CA- group.

Most of the mortality occurred during the first day, before assigning the bees to groups. The average survival rate for the first day in the three experiments detailed below was 50% for the allatectomized (*'CA-'*) bees, 80% for the sham, and 100% for the control bees. Survival of the bees after placed in groups was checked daily on days two to five. The recorded survival during these days was: 86% (45/52) for the CA- and CA-+JH, 94% (34/36) for the sham, and 100% (36/36) for the control bees. Only groups in which all three bees survived for the whole experiment were used for RNAseq analysis in order to keep the social state similar across groups. The bees were flesh frozen in liquid nitrogen on day five, and immediately transferred into marked 2 ml tubes that were immersed in dry ice. The tubes were later transferred to a deep freezer (-80ºC) until further analysis. This procedure assured that the bee samples are deeply frozen from the time of collection until the time of RNA extraction.

Assessing genetic variation within a colony

To estimate the genetic variability in our samples, we applied the GATK pipeline to the DNA-seq data of four worker bees that were used for the RNA-seq analyses. Average coverage depths in these four DNA-seq samples were 32, 32, 39 and 34X. More than 96% of the genome (236/245 Mbps) was covered by at least one sample. The average number of single nucleotide variants (SNVs) with respect to the reference genome was 1,286,561 (1,268,774-1,322,571), where most of these, 717,255 (56%), were common to all four bees, likely representing genetic variation between the Israeli *B. terrestris* population used in our study and the individual used to generate the official *B. terrestris* genome draft. Only 3-15% (36,223-193,007) of the variants were unique to a single bee. These results agree with our expectation that the four bee genomes are much closer to each other than to the reference genome. In total, 1,906,220 single nucleotide variants (SNVs) were detected (Supplementary Figure 3).

Supplementary Notes

Supplementary Note 1. Identification and characterization of the ADAR gene in the genomes of *B. terrestris* and *A. mellifera*

The *B. terrestris* ADAR ortholog was localized to coordinates 2874725-2893759 on the forward strand of chromosome 15.5 (using the Bter1.0 genome version for *B. terrestris*) [5]. We found two predicted mRNA isoforms: XM_012316883.1 (Isoform 1), and XM_012316884.1 (Isoform 2). These two isoforms are splice variants differing by sequence in the 5' UTR region (Supplementary Figure 1A). The predicted BtADAR gene products are XP_012172274.1 and XP_012172273.1. The *A. mellifera* ADAR ortholog was localized to chromosome 15.19 on coordinates 752501-755242 of the reverse strand (using genome version 4.5 [6]). The AmADAR gene produces two splice variants: XM_006563327.1 (Isoform 1) and XM_006563326.1 (Isoform 2), differing in the last exon (Supplementary Figure 1B). The AmADAR protein products are XP_006563389.1 (Isoform 1) & XP_006563390.1 (Isoform 2).

Supplementary Note 2. The majority of well-edited and well-covered hyper-editing sites are actually genomic polymorphisms

Hyper-editing analysis on 58 bee samples detected 1,150,394 unique genomic sites with very high specificity (only 0.96% of all non A-to-G mismatches), of these 208,089 sites (18%) reside in annotated coding regions (NCBI RefSeq annotations). Profiling the editing level across the 58 samples, we found that only 253 of these coding sites (0.12%) have a median coverage of at least 50 reads and a median editing level that exceeds 5%. We analyzed the editing level distributions of these 253 sites, and found that 170 are probably genomic polymorphisms rather than editing sites. Among these 170 sites the editing levels (i.e no. Gs/(no. Gs + no. As)) are either above 95%, or below 5%, and range between 30% to 70% in all the 58 samples.

Supplementary Note 3. Comparison of ADAR autoediting in the bumblebee to that in mice and flies

In mice, autoediting of ADAR2 alters the splicing pattern, resulting in a frame-shift and a premature stop codon, thus acting as a negative regulator of editing activity [7]. In Drosophila, autoediting results in recoding the ADAR amino acid sequence. The edited and unedited DmADAR isoforms target the same editing sites, however there are slightly different levels, with the edited enzyme showing reduced editing activity *in vivo* [8,9]. It is thought that the reduced activity of the edited form stems from a combination of altered catalytic function [10], and sequestration of auto-edited DmADAR proteins to a nuclear sub-compartment, resulting in reduced active concentration of DmADAR at transcription sites [9]. In the bumblebee, autoediting also results in recoding, but of a different amino acid than in Drosophila, and thus the effect on ADAR is not expected to be the same.

Supplementary Figures

A.

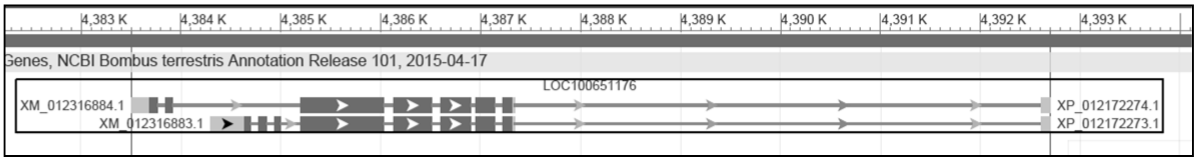

B.

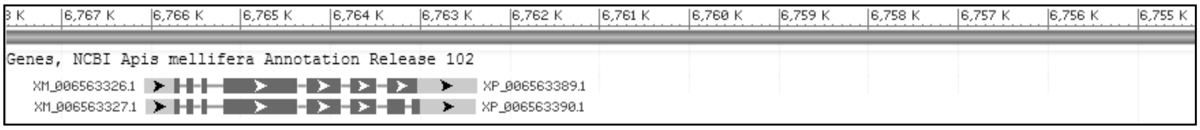

Supplementary Figure 1. Genomic models for ADAR orthologs in bees. **A**. *Bombus terrestris* (NCBI; Bter1.0 genome version). **B**. *Apis mellifera* (NCBI; genome version 4.5). Thick dark rectangles represent exons, thick light rectangles represent UTRs and thin lines represent introns.

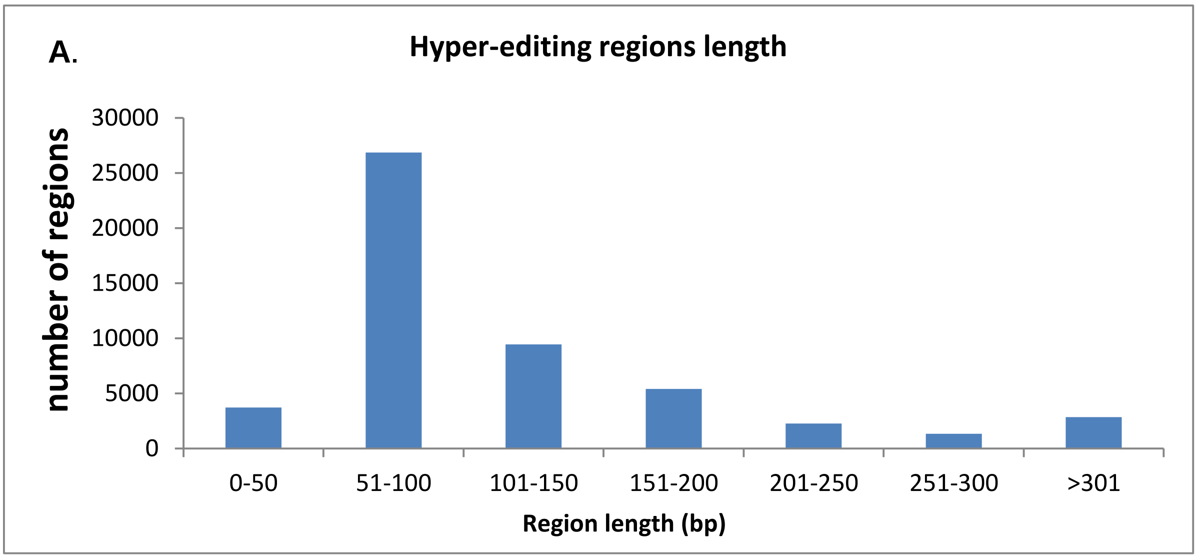

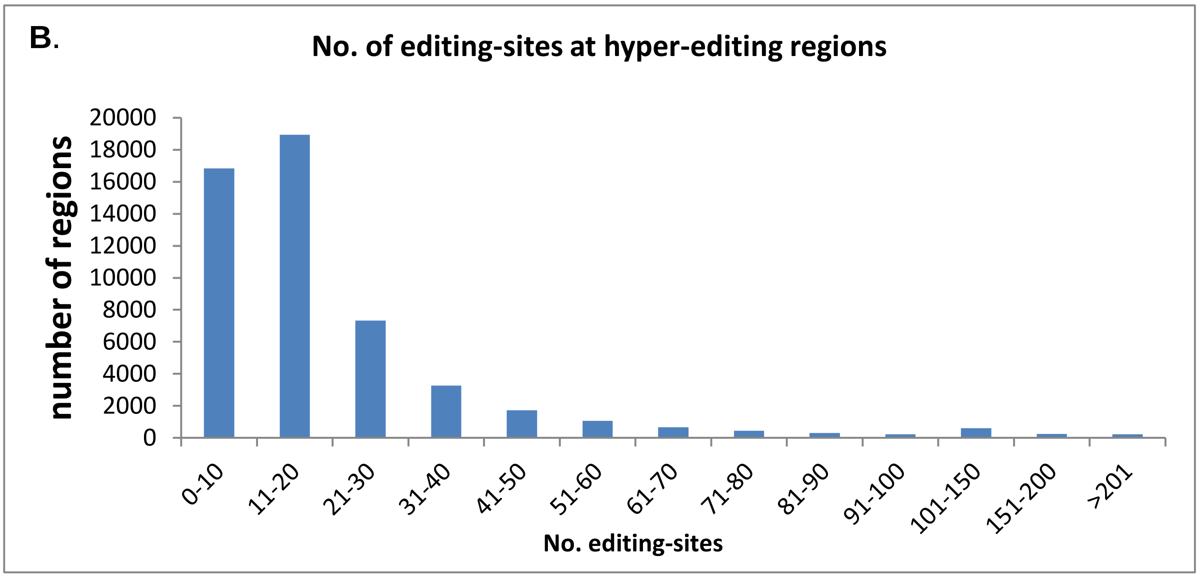

Supplementary Figure 2. Frequency distribution for sites with hyper-editing in the brain transcriptome of *Bombus terrestris* bumblebees. RNA-seq data was obtained from brain samples of 58 individual workers, consisting of ~4 billion 2x100bp reads. We identified 1,150,394 unique genomic sites that cluster to 51,930 distinct genomic regions. Average length of RNA edited clusters was 128.3bp ± 118.8 (**A**) and contained 22.2 ± 29.1 editing-sites (**B**).

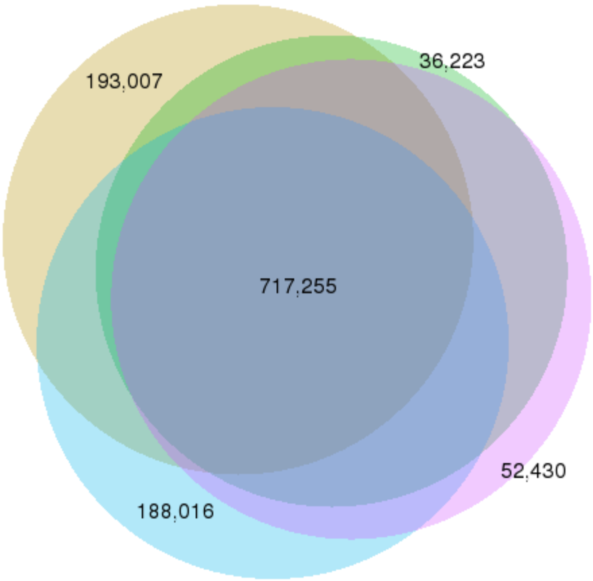

Supplementary Figure 3. Vann diagram representing the genetic variation within our bumblebee samples. Each circle depicts single nucleotide variants (SNVs) with respect to the reference genome in each one of the four genome sequenced bees using the GATK pipeline. In total, we identified 1,906,220 SNVs, with the number of SNVs ranging between 1,268,774 to 1,322,571 per bee. Most of these SNVs (717,255; 56%), were common to all four bees (represented by the overlapping area in the middle of the four circles). Only 3-15% (36,223-193,007) of the SVNs are unique to a single bee (areas with no overlap). These findings are consistent with the premise that these four bee genomes are more similar to each other than to the reference genome.

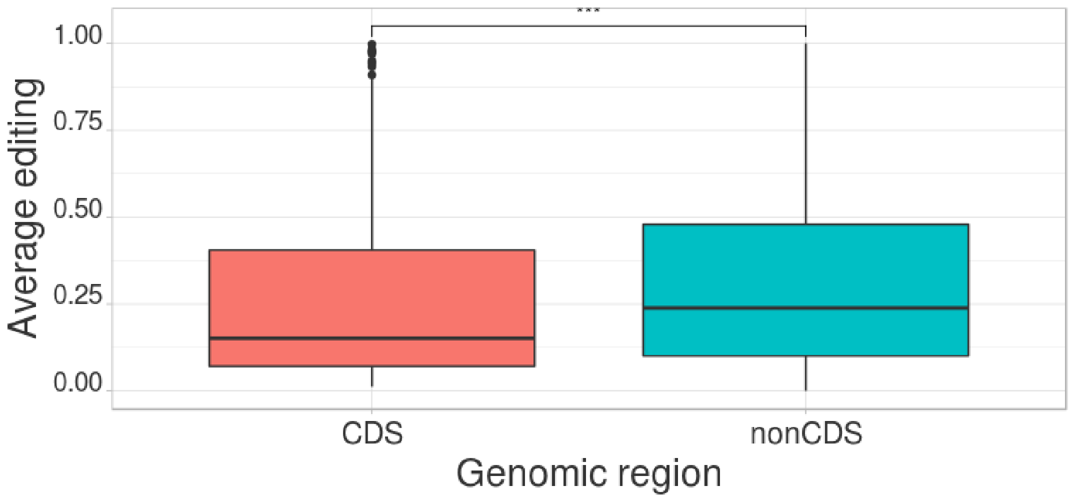

Supplementary Figure 4. Boxplot showing the distributions of editing levels (averaged over all 58 individual bees) for the 8340 editing sites detected using the MuTect pipeline. The level of editing in the 219 coding sites (CDS) is low compared to all other sites (Mann Whitney test, p-value=4.95E-04). Boxplots are a standardized method of displaying the distribution of data based on “minimum” [Q1-1.5*IQR], first quartile (Q1), median, third quartile (Q3), “maximum” [Q3+1.5*IQR] and outliers (where IQR is the interquartile range).

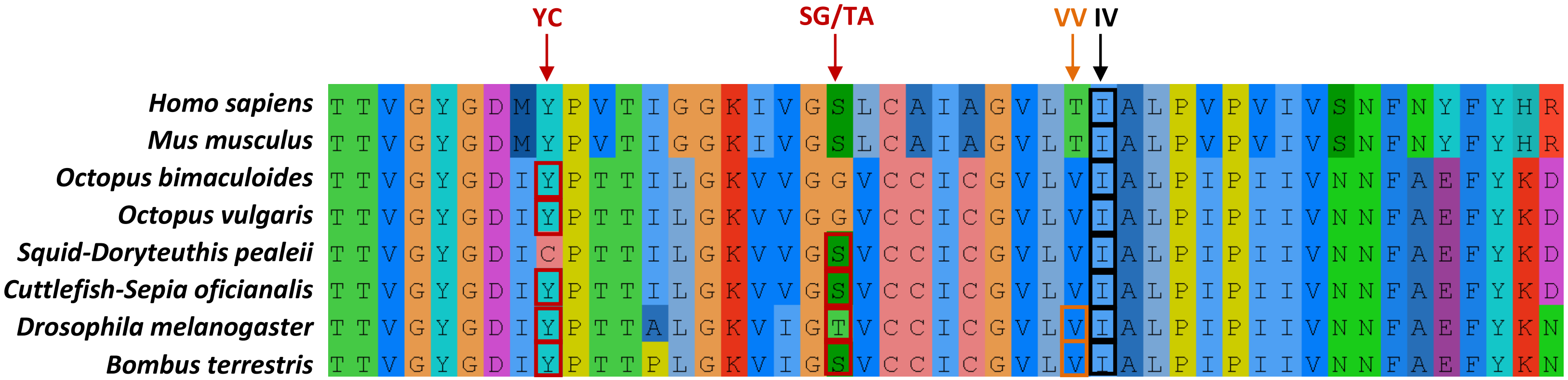

Supplementary Figure 5. Multiple sequence alignment of the amino acid sequence models of the potassium voltage-gated channel Shab protein of eight species. The four conserved editing sites within *B. terrestris* and *D. melanogaster* are denoted with arrows. Amino-acid replacements resulting from editing, are designated above the arrows, and the amino-acids that are prone to editing are denoted with squares. The red arrows point to editing sites that are also conserved in cephalopods and the black arrow points to the editing site that is also conserved in mammals.

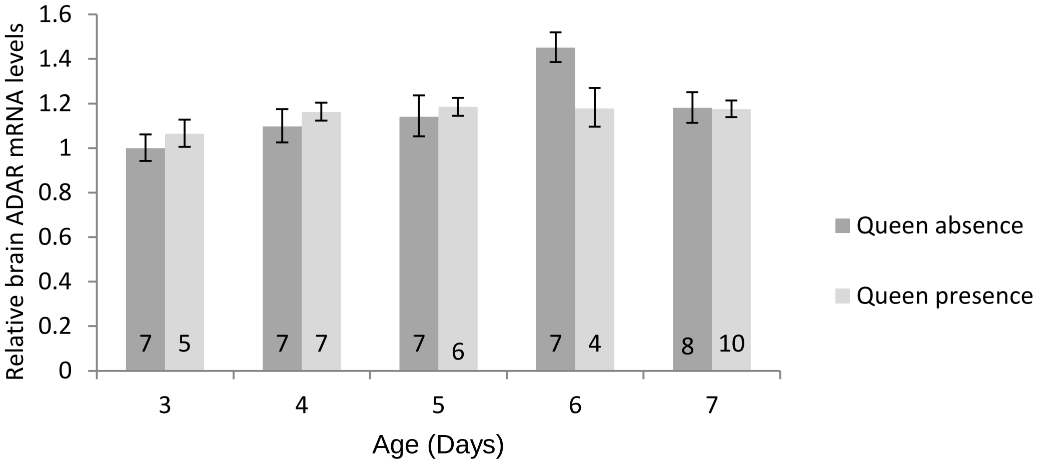

Supplementary Figure 6. Relative brain btADAR transcript abundance in bumblebee workers at various ages collected from queenless (Queen absence, dark grey bars) or queenright (Queen presence; light grey) colonies. Values are presented as mean ± SE. Sample size is shown inside each column.

Supplementary references

1. Hornáková, D., Matousková, P., Kindl, J., Valterová, I. & Pichová, I. Selection of reference genes for real-time polymerase chain reaction analysis in tissues from Bombus terrestris and Bombus lucorum of different ages. *Anal. Biochem.* **397,** 118–20 (2010).
2. Vandesompele, J. *et al.* Accurate normalization of real-time quantitative RT-PCR data by geometric averaging of multiple internal control genes. *Genome Biol.* **3,** RESEARCH0034 (2002).
3. Andersen, C. L., Jensen, J. L. & Ørntoft, T. F. Normalization of Real-Time Quantitative Reverse Transcription-PCR Data: A Model-Based Variance Estimation Approach to Identify Genes Suited for Normalization, Applied to Bladder and Colon Cancer Data Sets. *Cancer Res.* **64,** 5245–5250 (2004).
4. Pfaffl, M. W., Tichopad, A., Prgomet, C. & Neuvians, T. P. Determination of stable housekeeping genes, differentially regulated target genes and sample integrity: BestKeeper - Excel-based tool using pair-wise correlations. *Biotechnol. Lett.* **26,** 509–515 (2004).
5. Sadd, B. M. *et al.* The genomes of two key bumblebee species with primitive eusocial organization. *Genome Biol.* **16,** 76 (2015).
6. Munoz-Torres, M. C. *et al.* Hymenoptera Genome Database: integrated community resources for insect species of the order Hymenoptera. *Nucleic Acids Res.* **39,** D658-62 (2011).
7. Rueter, S. M., Dawson, T. R. & Emeson, R. B. Regulation of alternative splicing by RNA editing. *Nature* **399,** 75–80 (1999).
8. Palladino, M. J., Keegan, L. P., O'Connell, M. A. & Reenan, R. A. dADAR, a Drosophila double-stranded RNA-specific adenosine deaminase is highly developmentally regulated and is itself a target for RNA editing [In Process Citation]. *Rna* **6,** 1004–1018 (2000).
9. Savva, Y. A. *et al.* Auto-regulatory RNA editing fine-tunes mRNA re-coding and complex behaviour in Drosophila. *Nat. Commun.* **3,** 790 (2012).
10. Keegan, L. P. *et al.* Tuning of RNA editing by ADAR is required in Drosophila. *EMBO J.* **24,** 2183–2193 (2005).
