# Supplementary material for: RNA editing is abundant and correlates with task performance in a social bumblebee
Source: Nat Commun. 2019 Apr 8;10:1605. doi: 10.1038/s41467-019-09543-w (PMC6453909; doi:10.1038/s41467-019-09543-w)
Supplement: Supplementary file 3 — Reporting Summary [file 41467_2019_9543_MOESM3_ESM.pdf]

nature research

Corresponding author(s): Guy Bloch (guy.bloch@mail.huji.ac.il)

Reporting Summary

Nature Research wishes to improve the reproducibility of the work that we publish. This form provides structure for consistency and transparency in reporting. For further information on Nature Research policies, see Authors & Referees and the Editorial Policy Checklist.

Statistical parameters

When statistical analyses are reported, confirm that the following items are present in the relevant location (e.g. figure legend, table legend, main text, or Methods section).

n/a	Confirmed	
☐	☒	The exact sample size (n) for each experimental group/condition, given as a discrete number and unit of measurement
☐	☒	An indication of whether measurements were taken from distinct samples or whether the same sample was measured repeatedly
☐	☒	The statistical test(s) used AND whether they are one- or two-sided<br>*Only common tests should be described solely by name; describe more complex techniques in the Methods section.*
☒	☐	A description of all covariates tested
☐	☒	A description of any assumptions or corrections, such as tests of normality and adjustment for multiple comparisons
☐	☒	A full description of the statistics including central tendency (e.g. means) or other basic estimates (e.g. regression coefficient) AND variation (e.g. standard deviation) or associated estimates of uncertainty (e.g. confidence intervals)
☐	☒	For null hypothesis testing, the test statistic (e.g. F, t, r) with confidence intervals, effect sizes, degrees of freedom and P value noted<br>*Give P values as exact values whenever suitable.*
☒	☐	For Bayesian analysis, information on the choice of priors and Markov chain Monte Carlo settings
☒	☐	For hierarchical and complex designs, identification of the appropriate level for tests and full reporting of outcomes
☒	☐	Estimates of effect sizes (e.g. Cohen's d, Pearson's r), indicating how they were calculated
☐	☒	Clearly defined error bars<br>*State explicitly what error bars represent (e.g. SD, SE, CI)*

Our web collection on statistics for biologists may be useful.

Software and code

Policy information about availability of computer code

Data collection	No software was used in order to collect the data
Data analysis	Hyper-editing scripts (from https://www.nature.com/articles/ncomms5726 availble at https://github.com/hagitpt/Hyper-editing)<br>STAR aligner (2.3), BWA (version 0.6.2), Picard (version 1.70), GATK (version 3.1.1), MuTect (version 1.1.4), REDItools (version 1.0.3)

For manuscripts utilizing custom algorithms or software that are central to the research but not yet described in published literature, software must be made available to editors/reviewers upon request. We strongly encourage code deposition in a community repository (e.g. GitHub). See the Nature Research guidelines for submitting code & software for further information.

Data

Policy information about availability of data

All manuscripts must include a data availability statement. This statement should provide the following information, where applicable:
- Accession codes, unique identifiers, or web links for publicly available datasets
- A list of figures that have associated raw data
- A description of any restrictions on data availability

The accession code for all the sequencing data that was used in the article is PRJNA497863.
There are no figures that have associated raw data, and no restrictions on data availability.

Field-specific reporting

Please select the best fit for your research. If you are not sure, read the appropriate sections before making your selection.

☒ Life sciences ☐ Behavioural & social sciences ☐ Ecological, evolutionary & environmental sciences

For a reference copy of the document with all sections, see nature.com/authors/policies/ReportingSummary-flat.pdf

Life sciences study design

All studies must disclose on these points even when the disclosure is negative.

Sample size	Given the cost of RNA sequencing and DNA sequencing we limited the sample size to up to 8 and 4 bees per experimental group, respectively. For the qPCR and Snager we used larger sample sizes which were determined post-hoc base on the results we obtained.
Data exclusions	We excluded Sanger sequencing data and qPCR amplifications that did not work properly. Given that we run the qPCR in triplicates, in most cases this did not require excluding a sample, but rather calculating the average based on 2 rather than 3 technical replicates.
Replication	We repeated the RNA sequencing experiments only once. Some qPCR and Sanger sequencing experiments were repeated with bees from more than one source colony and the results of the different trials are reported.
Randomization	In the JH manipulation experiment bees were assigned randomly to treatment. In the other experiments bees were not assigned to treatment but rather compared based on the behavior they showed (i.e., foraging, brood care, dominance rank)
Blinding	The analyses performed here (DNA and RNA sequencing, qPCR, etc are blind to the treatment.

Reporting for specific materials, systems and methods

Materials & experimental systems

n/a	Involved in the study
☒	☐ Unique biological materials
☒	☐ Antibodies
☒	☐ Eukaryotic cell lines
☒	☐ Palaeontology
☐	☒ Animals and other organisms
☒	☐ Human research participants

Methods

n/a	Involved in the study
☒	☐ ChIP-seq
☒	☐ Flow cytometry
☒	☐ MRI-based neuroimaging

Animals and other organisms

Policy information about studies involving animals; ARRIVE guidelines recommended for reporting animal research

Laboratory animals	bumblebee colonies were purchased from commercial vendors and maintained under standard conditions
Wild animals	*Provide details on animals observed in or captured in the field; report species, sex and age where possible. Describe how animals were caught and transported and what happened to captive animals after the study (if killed, explain why and describe method; if released, say where and when) OR state that the study did not involve wild animals.*
Field-collected samples	*For laboratory work with field-collected samples, describe all relevant parameters such as housing, maintenance, temperature, photoperiod and end-of-experiment protocol OR state that the study did not involve samples collected from the field.*
