# Supplementary material for: RNA editing is abundant and correlates with task performance in a social bumblebee
Source: Nat Commun. 2019 Apr 8;10:1605. doi: 10.1038/s41467-019-09543-w (PMC6453909; doi:10.1038/s41467-019-09543-w)
Supplement: Supplementary file 4 — Description of Additional Supplementary Files [file 41467_2019_9543_MOESM4_ESM.docx]

**Description of Additional Supplementary Files**

File Name: Supplementary Data 1

Description: 208,089 coding hyper-edited sites

File Name: Supplementary Data 2

Description: 8340 genome wide editing sites detected by MuTect

File Name: Supplementary Data 3

Description: 219 edited coding sites

File Name: Supplementary Data 4

Description: 100 edited coding genes

File Name: Supplementary Data 5

Description: GO analysis results of the 100 edited coding genes

File Name: Supplementary Data 6

Description: 149 well-covered edited coding sites

File Name: Supplementary Data 7

Description: 7 recoding editing sites conserved between bee and flies

File Name: Supplementary Data 8

Description: 14 coding sites that are correlate with auto-editing

File Name: Supplementary Data 9

Description: 13 coding sites that are differentially edited in N and F
