# Supplementary material for: RNA editing is abundant and correlates with task performance in a social bumblebee
Source: Nat Commun. 2019 Apr 8;10:1605. doi: 10.1038/s41467-019-09543-w (PMC6453909; doi:10.1038/s41467-019-09543-w)
Supplement: Supplementary file 2 — Peer Review File [file 41467_2019_9543_MOESM2_ESM.pdf]

Reviewers' comments:

Reviewer #1 (Remarks to the Author):

Summary

The submitted study explored the extent of RNA editing in bumble bee tissues and in bees involved in different behavioral activities. They found higher levels of editing than expected given what was found previously in Drosophila. As expected, editing was higher in the brain and in RNAs that form double stranded structures. The evidence for editing effects on any aspect of social behavior was weak, suggesting that the editing serves more basic cellular functions. The writing of the paper needs work and some aspects of the study are not well explained, but overall it is a solid contribution to the literature.

Questions/suggestions for improvement

Although editing has been explored in an ant there was little to no discussion of what was found in ants relative to what was found here. I think a section in the discussion on this would be useful.

I don't know much about the bioinformatics of identifying editing sites, and given how recent the cited papers are, I imagine most others who will read the paper also don't. Given this, some more discussion of exactly how one differentiates between variation (SNPs) and editing between the genome sequence and the RNA seq reads would be helpful. There seems to be a disconnect between what is found in that section and what is found when looking at the new Bombus DNA reads but perhaps that is just because of an omission of some details?

The writing is okay, but not great throughout. If no one on the paper is an English native speaker it might be useful to have a collaborator do some simple copy editing to improve the readability.

Line 47: missing parentheses end

Lines 211 to 239: the paper has many very long unorganized paragraphs. This one for example has several distinct topics and actually switches from bumble bees to honey bees without starting a new paragraph

Reviewer #2 (Remarks to the Author):

In this work the authors investigated the A-to-I RNA editing in the bumblebee for the first time and they revealed extensive RNA editing in this insect species. Despite bumblebee does not have ADAR1, the normalized hyper-editing signal (A-to-G mismatches per million mapped bases) in the bumblebee is higher than that in human or mouse brain. They further found 164 recoding sites residing in 100 protein-coding genes, some of which are conserved across insects, cephalopods and mammals. These findings are interesting, but there are still some questions remaining to be answered.

1. Both the hyper-editing sites and single editing sites should be characterized in more detail. E.g. the proportion of these sites in 5'UTR, CDS, 3'UTR and intron; the distribution of editing levels for different groups of editing sites. These descriptions would be helpful for us to understand the possible biological functions of these editing events.

2. What are the repeat features of both hyper-editing sites and single editing sites? It is interesting to see a large number of hyper-editing events in bumblebee, so the authors should look into this in detail

to reveal the possible reason. Is there any repeat (such as Alus in human) that is mainly targeted by ADAR in this species?

3. Compared with D.mel, bumblebee has much less recoding sites. However, it has a lot more hyper-editing sites. What kind of genomic feature difference between these two insect species may lead to such editing difference?

4. I did not find the positive correlation between editing levels of sites located in ADAR gene and the overall editing level a very interesting analysis. As the overall editing levels increase, the editing level of any individual site may increase. In addition, how about the correlation between ADAR expression and ADAR auto-editing?

5. The relationship between RNA editing and task performance is not very convincing since the correlation is weak. The authors may need to tone down the conclusions.
Minor points:

1. Line 345: The subtitle "The B. terrestris transcriptome is extensively edited" is not necessary.
2. Line 355: "In average, an RNA edited cluster was 128bp long and contained 22 editing-sites". It's better to provide a figure to show both the length distribution of edited clusters and the distribution of editing site number per edited cluster.

3. Line 390: The reference number of MuTect tool is wrong. It is not ref 66 "Development of Different Analysis Platforms with LC−MS for Pharmacokinetic Studies of Protein Drugs" but ref 59 "Sensitive detection of somatic point mutations in impure and heterogeneous cancer samples".

Reviewer #3 (Remarks to the Author):

Porath et al. investigated A-to-I RNA editing in bumblebee and found that RNA editing is abundant and correlates with task specialization. They found a higher editing signal in bumblebee than in fly and even in human. They suggested that bumblebee ADAR enzyme (and probably that of other insects) can take some of the functions that in mammals are performed by ADAR1 and by that compensate to some extent for the lack of a second editing enzyme. Brain editing levels are high in naturally behaving bees and correlated with task nursing/foraging activity. This is an interesting study. I have several suggestions as follows.

Major points:
1. When detecting RNA editing sites using comparisons of DNA-seq and RNA-seq, the authors chose MuTect, which was developed to detect somatic mutations in cancer samples. It would be better to explain/discuss why the authors use this tool to identify RNA editing sites? For example, REDItools, RES-scanner, JACUSA, and RDDpred are all based on RNA-DNA comparison (recently reviewed by one of the authors (Pinto, Y., & Levanon, E. Y., 2018, Methods)).

2. (Figure 1A, B) Since the regular-edited and hyper-edited reads were called by different methods, the editing level distribution should be analyzed and discussed.

3. (Figure 1C) The authors found that the editing signal of bumblebee was higher than fruit fly, even higher than human brain signal. It would be better to examine the editing signal in honey bee, ant (e.g., leaf-cutting ant) and other Drosophila species. The authors can functional analyze the

bumblebee-specific edited genes. In addition, are the editing levels of recoding sites shared by different insects highly correlated with each other?

4. The authors suggested that bumblebee ADAR enzyme (and probably that of other insects) can take some of the functions that in mammals are performed by ADAR1 and by that compensate to some extent for the lack of a second editing enzyme. The authors should explore the correlation between editing events (clustered editing events especially) and transposon elements in bumblebee.

5. (Figure 2) The diagram of Shab voltage gated ion channel shows a lot of editing sites on the 6th transmembrane domain and N-terminus inner cytoplasmic domain. Is it possible that this transcript of RNA structure contains the "editing induced element" (Daniel, C., Widmark, A., Rigardt, D., & Öhman, M. (2017). Genome biology, 18(1), 195.)?

6. (Figure 3) The level of auto-editing of BtADAR is correlated with average editing level. It is interesting to further analyze the specific sites/genes that are highly correlated with auto-editing of BtADAR.
7. (Figure 4A-D) The worker bees can be classified into two groups, nurses and foragers, in terms of editing level. I am curious about whether the similar result holds when considering the expression levels of the corresponding edited genes?
8. In terms of evolution, the authors can explore social insect-specific editing sites or edited genes (based on two social insects bumblebee and leaf-cutting ant), and the possible corresponding functions.

Minor points:
9. The reference [66] seems to be incorrect annotated, the reviewer thinks the original one may be "Cibulskis, Kristian, et al. Nature biotechnology 31.3 (2013): 213."
10. Figure 2 legend: the word "transmembrance" may be "transmembrane".
11. Figure 6B-E: the y-axis should be labeled to avoid misunderstanding.

Below is our point by point response to all comments raised by the reviewers.

Reviewers' comments:

**Reviewer #1:**

Summary

The submitted study explored the extent of RNA editing in bumble bee tissues and in bees involved in different behavioral activities. They found higher levels of editing than expected given what was found previously in Drosophila. As expected, editing was higher in the brain and in RNAs that form double stranded structures. The evidence for editing effects on any aspect of social behavior was weak, suggesting that the editing serves more basic cellular functions. The writing of the paper needs work and some aspects of the study are not well explained, but overall it is a solid contribution to the literature.

Questions/suggestions for improvement

Although editing has been explored in an ant there was little to no discussion of what was found in ants relative to what was found here. I think a section in the discussion on this would be useful.
The Introduction and the Discussion both include explicit reference to the ants study. Following the reviewer's comment, we extended the corresponding section in the Discussion, to allow for a better comparison with our results. It now reads as follows (newly added sentences underlined): "The only previous study on RNA editing in social insects compared morphologically distinct castes of the highly social leaf-cutting ant *Acromyrmex echinatior*. <u>This study reported a consistent editing pattern for each of the three castes considered (gynes, large workers, small workers), but these sites recoded overall only 34 protein coding genes, only three of these are also edited in other ant species that they tested.</u> The differences between queen and worker ants are considerable, many of which are determined during development, and are fixed for life. Here we asked whether RNA editing can be associated with relatively short-term changes during the adult stage of bumblebee workers. We found that brain RNA editing was not influenced by dominance rank (which is associated with aggressiveness) or JH (which regulates fertility and aggression in bumblebees) manipulation, but was correlated with the task the worker bee performs. <u>Similarly to the ants, while the global editing pattern could be correlated with behavior, very few individual sites could be pinpointed as being modified."</u>

I don't know much about the bioinformatics of identifying editing sites, and given how recent the cited papers are, I imagine most others who will read the paper also don't. Given this, some more discussion of exactly how one differentiates between variation

(SNPs) and editing between the genome sequence and the RNA seq reads would be helpful.

We added two short explanatory sentences in the Results section on hyper-editing, and a reference to a recent review paper (new text underlined): "To find clustered editing sites, we applied the hyper-editing detection scheme. This approach looks for multiple consecutive mismatches of the same type between the reference genome and the RNA-seq data (e.g. genomic-A:RNA-G), which are unlikely to result from genomic polymorphisms" and in the next section: "In order to look for isolated DNA-RNA mismatches, we sequenced the DNA of four of the 58 bees, aligned the matched DNA and RNA data to the genome, and used the MuTect tool to identify 9653 reliable DNA-RNA mismatches. Similar to many other schemes for editing detection, this tool looks for consistent mismatches between the RNA-seq reads and the reference genome, for which the DNA-seq reads do not show any sign of variation."

There seems to be a disconnect between what is found in that section and what is found when looking at the new Bombus DNA reads but perhaps that is just because of an omission of some details?

We added the following explanation regarding the difference between the two methods: "This method is less sensitive to clusters of editing sites, as it misses many clusters due to low DNA-seq coverage, for example. However, it allows for detection of editing sites that are not part of a cluster, and results in a set of editing sites that are (on average) more highly expressed and more highly edited than those found by the hyper-editing scheme. ".

The writing is okay, but not great throughout. If no one on the paper is an English native speaker it might be useful to have a collaborator do some simple copy editing to improve the readability.

We carefully reviewed the manuscript again, and improved the writing, including editing by a native English speaker as suggested by the reviewer.

Line 47: missing parentheses end

Fixed

Lines 211 to 239: the paper has many very long unorganized paragraphs. This one for example has several distinct topics and actually switches from bumble bees to honey bees without starting a new paragraph

We moved the experiment with honeybees to a new paragraph, and improved the writing of this section. We hope that in its current form this section clearly conveys our experimental procedure.

**Reviewer #2:**

In this work the authors investigated the A-to-I RNA editing in the bumblebee for the first time and they revealed extensive RNA editing in this insect species. Despite bumblebee does not have ADAR1, the normalized hyper-editing signal (A-to-G mismatches per million mapped bases) in the bumblebee is higher than that in human or mouse brain.

They further found 164 recoding sites residing in 100 protein-coding genes, some of which are conserved across insects, cephalopods and mammals. These findings are interesting, but there are still some questions remaining to be answered.

1. Both the hyper-editing sites and single editing sites should be characterized in more detail. E.g. the proportion of these sites in 5’UTR, CDS, 3’UTR and intron; the distribution of editing levels for different groups of editing sites. These descriptions would be helpful for us to understand the possible biological functions of these editing events.
We certainly agree that better annotation of the sites would be helpful. However, the transcriptome of the bumble bee is not well characterized at present to allow for this analysis. Coding sequences were found based on open reading frames and conservation to other species, however, the UTRs are not really known at a reasonable accuracy (most reported transcripts have very short UTRs, suggesting they are incomplete). Thus, unfortunately, it is not possible to classify sites reliably as UTRs vs. intergenic regions.

Accordingly, we only calculated distribution of editing levels for CDS and non-CDS sites (added to the paper as Supp. Fig. S4).

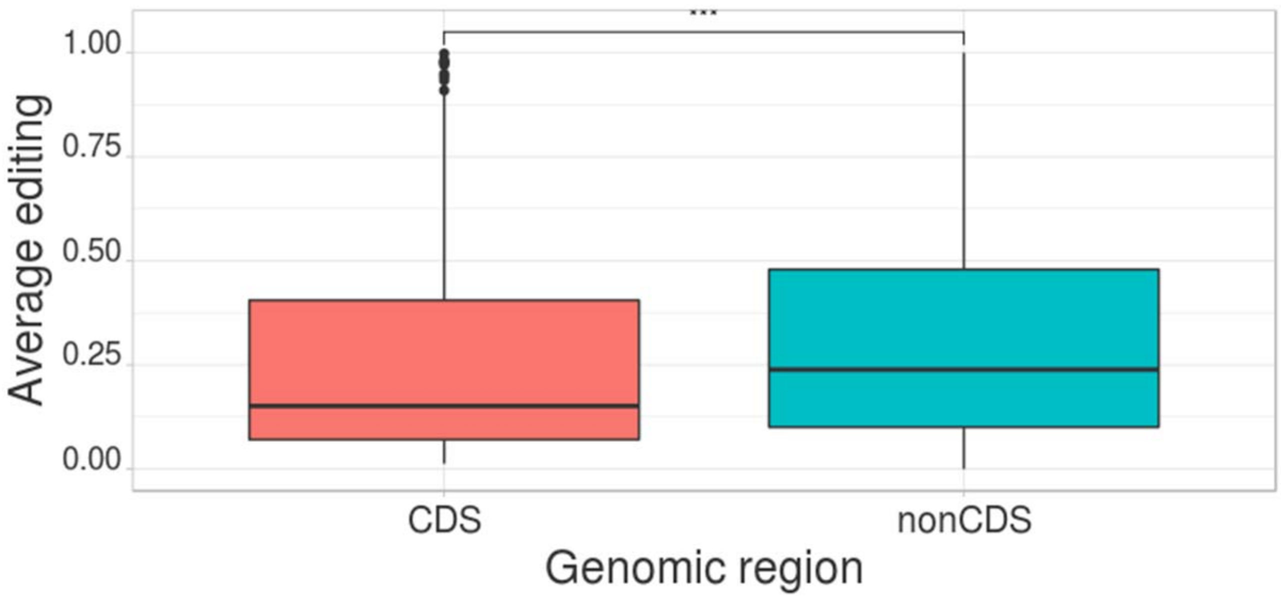


2. What are the repeat features of both hyper-editing sites and single editing sites? It is interesting to see a large number of hyper-editing events in bumblebee, so the authors should look into this in detail to reveal the possible reason. Is there any repeat (such as Alus in human) that is mainly targeted by ADAR in this species?
Here too, we agree this is a very important issue to explore, but unfortunately the available data on repetitive elements in the current bumblebee genome version,

https://www.ncbi.nlm.nih.gov/genome/annotation_euk/Bombus_terrestris/102/ does not allow for this analysis.

Instead, we looked at a related question - the correlation of editing sites with long and stable dsRNAs. In the first version, we showed that the detected hyper-edited regions are enriched in putative dsRNA structures ("*A-to-I RNA hyper editing events*" section at the *Results*). In the revised manuscript we have expanded this analysis, adding a control set of random regions with the same lengths as the tested HE regions. We have added to the paper the following: "Furthermore, putative dsRNAs were found for only 1.6% (73/4510) of random regions of similar length."

3. Compared with D.mel, bumblebee has much less recoding sites. However, it has a lot more hyper-editing sites. What kind of genomic feature difference between these two insect species may lead to such editing difference?

We assume that the main reason for the difference between the two insects is the abundance of dsRNA structures (see Porath et al, ref. 34). One factor contributing to this abundance is the fraction of repetitive elements in the genome. The difference in genome size (drosophila – 138Mbp; bumble bee – 249Mb) may indeed suggest a higher prevalence of repetitive elements in the bumble bee. A detailed analysis of this hypothesis is not possible at this point of time, as explained above, due to the lack of reliable annotation of the bumblebee UTRs and repetitive elements. We hope to be able to study this in the future.

4. I did not find the positive correlation between editing levels of sites located in ADAR gene and the overall editing level a very interesting analysis. As the overall editing levels increase, the editing level of any individual site may increase. In addition, how about the correlation between ADAR expression and ADAR auto-editing?

We agree that the explanation for the observed correlation may very well be the overall increase in editing activity. We have toned down the text to help clarify this point. We also mention in the revised text that none of the three auto-editing sites was found to be significantly correlated with ADAR expression level (p-values 0.47, 0.36 and 0.31, respectively).

5. The relationship between RNA editing and task performance is not very convincing since the correlation is weak. The authors may need to tone down the conclusions.

We amended the text in a number of places, to tone down our conclusions. For example, the Discussion now reads: "Admittedly, the association between task performance and RNA editing in the brain is rather weak. However, task specialization in bumblebees is also relatively weak and flexible, with many workers switching between brood care and foraging activities within the same day."

Minor points:

1. Line 345: The subtitle "The B. terrestris transcriptome is extensively edited" is not necessary.

We removed this title and moved the following introductory text below the next title.

2. Line 355: "In average, an RNA edited cluster was 128bp long and contained 22 editing-sites". It's better to provide a figure to show both the length distribution of edited clusters and the distribution of editing site number per edited cluster.
The requested distributions are now shown in the new Supp. Figure S2.

3. Line 390: The reference number of MuTect tool is wrong. It is not ref 66 "Development of Different Analysis Platforms with LC−MS for Pharmacokinetic Studies of Protein Drugs" but ref 59 "Sensitive detection of somatic point mutations in impure and heterogeneous cancer samples".
Fixed.

**Reviewer #3:**

Porath et al. investigated A-to-I RNA editing in bumblebee and found that RNA editing is abundant and correlates with task specialization. They found a higher editing signal in bumblebee than in fly and even in human. They suggested that bumblebee ADAR enzyme (and probably that of other insects) can take some of the functions that in mammals are performed by ADAR1 and by that compensate to some extent for the lack of a second editing enzyme. Brain editing levels are high in naturally behaving bees and correlated with task nursing/foraging activity. This is an interesting study. I have several suggestions as follows.

Major points:

1. When detecting RNA editing sites using comparisons of DNA-seq and RNA-seq, the authors chose MuTect, which was developed to detect somatic mutations in cancer samples. It would be better to explain/discuss why the authors use this tool to identify RNA editing sites? For example, REDItools, RES-scanner, JACUSA, and RDDpred are all based on RNA-DNA comparison (recently reviewed by one of the authors (Pinto, Y., & Levanon, E. Y., 2018, Methods)).
We thank the reviewer for raising this point. In fact, we have tried to use REDItools (RNA-DNA mode), but did not end up with a reliable set of recoding sites (after some fine-tuning of the parameters, we got only up to 43% AG). This is not surprising, as most RNA-detection schemes do not perform very well in detecting recoding sites (see Ref. 21). Interestingly, MuTect was successful where most commonly-used schemes were not, leading to AG mismatches being 64% of all identified CDS mismatches. It might be worthwhile to explore the power of MuTect for editing detection in other contexts. We have added a sentence to the text, to highlight this point:
"Notably, the fraction of A-to-G sites among all mismatches identified by MuTect in coding regions was 64% (219/341), providing a signal-to-noise ratio much better than that obtained by standard RNA-editing detection schemes."

2. (Figure 1A, B) Since the regular-edited and hyper-edited reads were called by different methods, the editing level distribution should be analyzed and discussed.

The newly added Fig S4 provides the distributions of CDS and non-CDS editing levels (see response to Reviewer #2). In addition, the main text includes the following sentences: "Profiling the hyper-editing level in protein coding sites across the 58 samples, we found that only 253 (0.12%) have a median coverage of at least 50 reads and a median editing level exceeding 5%.", and

"The sites are well-covered (reads per site per sample: average 107 ± 102 (1 std), median 85), and the editing levels (averaged over the 58 bee samples) vary, with an average of 26% ± 27% (1 std) and median of 16%."

3. (Figure 1C) The authors found that the editing signal of bumblebee was higher than fruit fly, even higher than human brain signal. It would be better to examine the editing signal in honey bee, ant (e.g., leaf-cutting ant) and other Drosophila species. The authors can functional analyze the bumblebee-specific edited genes. In addition, are the editing levels of recoding sites shared by different insects highly correlated with each other?

We thank the referee for these excellent suggestions. Along with this comment we added the ant and honeybee to Figure 1C. They both show a signal comparable to that of human, and lower than the bumblebee. The editing levels of the fly-bee conserved sites are now reported in Supp. Table S9. Editing levels are similar for the two species.

4. The authors suggested that bumblebee ADAR enzyme (and probably that of other insects) can take some of the functions that in mammals are performed by ADAR1 and by that compensate to some extent for the lack of a second editing enzyme. The authors should explore the correlation between editing events (clustered editing events especially) and transposon elements in bumblebee.

Please see our response to Reviewer #2 (point 2). In the absence of high quality repeat annotation, we cannot look at this interesting aspect, but we did show that the hyper-edited clusters tend to occur in long dsRNA regions.

5. (Figure 2) The diagram of Shab voltage gated ion channel shows a lot of editing sites on the 6th transmembrane domain and N-terminus inner cytoplasmic domain. Is it possible that this transcript of RNA structure contains the "editing induced element" (Daniel, C., Widmark, A., Rigardt, D., & Öhman, M. (2017). Genome biology, 18(1), 195.)?

We thank the referee for this interesting suggestion. However, we could not find any hyper-editing cluster, not a long stable dsRNA (i.e. a reverse-complemented alignment) within their flanking ±2 kb genomic sequence.

6. (Figure 3) The level of auto-editing of BtADAR is correlated with average editing level. It is interesting to further analyze the specific sites/genes that are highly correlated with auto-editing of BtADAR.

Looking at specific editing sites, we found 14 of the 219 coding sites to be significantly and positively correlated with the I482M auto-edit site. This information was added to the

revised manuscript (it is mentioned in main text in p. 7 (first paragraph of "*Autoediting of BtADAR*"), and sites are reported in the newly added Supp. Table S10).

7. (Figure 4A-D) The worker bees can be classified into two groups, nurses and foragers, in terms of editing level. I am curious about whether the similar result holds when considering the expression levels of the corresponding edited genes?
The nurses and foragers for which we report here results on RNA editing can be nicely separated based on the pattern of transcript abundance. These results will be reported in a separate paper that currently in preparation which focuses on differentially expressed genes that are associated with task performance. Following the Reviewer's request, we specifically tested whether transcripts that are differentially edited in nurses and foragers also differ in their abundance. We found, with the exception of the glucose dehydrogenase [acceptor]-like (XM_003395620.1), that these transcripts do not differ in their abundance between nurses and foragers. We added this information to column AO of Supp. Table S11. We added a sentence referring to this analysis in p. 8 (last paragraph): "With the exception of one gene, these genes did not differ in their transcript abundance between nurses and foragers (see column AO in Supplementary Table S11).".

8. In terms of evolution, the authors can explore social insect-specific editing sites or edited genes (based on two social insects bumblebee and leaf-cutting ant), and the possible corresponding functions.
As explained in the main text, we cannot currently pinpoint specific editing sites that correlate with social behavior. All we see at this point is a global correlation. We hope that future research will uncover the subset of editing sites which are related to social behavior, and then one would be able to explore the functional meaning of these events and their connection to task performance.

Minor points:

9. The reference [66] seems to be incorrect annotated, the reviewer thinks the original one may be "Cibulskis, Kristian, et al. Nature biotechnology 31.3 (2013): 213."
Fixed

10. Figure 2 legend: the word "transmembrance" may be "transmembrane".
Fixed

11. Figure 6B-E: the y-axis should be labeled to avoid misunderstanding.
Done

REVIEWERS' COMMENTS:

Reviewer #1 (Remarks to the Author):

I am satisfied with the responses to my comments and the changes made in consequence.

Reviewer #2 (Remarks to the Author):

The authors have answered my questions satisfactorily.

Reviewer #3 (Remarks to the Author):

The authors have addressed most of the reviewer’s comments. The answers are generally acceptable. I only have several minor comments as follows.

1. The authors claimed that the AG% of MuTect is much higher than that of other similar detection schemes in coding regions. It would be better to provide a supplementary figure for comparisons of the accuracy (e.g., AG%) of these tools. The references of the related tools should be cited.
2. For the 14 coding editing sites that are significantly correlated with the editing level of the auto-editing site, the authors may functionally discuss the host genes of these editing events.
3. Although the authors are preparing a separated paper to explore differentially expressed genes, it would be better to perform PCA and examine whether the expression profile for the host genes of the editing sites in Fig. 4A can distinguish between the tasks of nurses and foragers.
4. Regarding Drosophila RNA editing in Introduction and “conserved recoding editing sites” in Method, some related studies can be cited, e.g., Cell Reports 11(5), 2015, 697-703; Genome Biology and Evolution 10(2), 2018, 521-537; and PNAS, 2019, pnas.1811768116.

REVIEWERS' COMMENTS:

Reviewer #1 (Remarks to the Author):

I am satisfied with the responses to my comments and the changes made in consequence.

Reviewer #2 (Remarks to the Author):

The authors have answered my questions satisfactorily.

Reviewer #3 (Remarks to the Author):

The authors have addressed most of the reviewer's comments. The answers are generally acceptable. I only have several minor comments as follows.

1. The authors claimed that the AG% of MuTect is much higher than that of other similar detection schemes in coding regions. It would be better to provide a supplementary figure for comparisons of the accuracy (e.g., AG%) of these tools. The references of the related tools should be cited.

** We added a sentence addressing this comment to the Methods section (p. 19, 1st paragraph)

2. For the 14 coding editing sites that are significantly correlated with the editing level of the auto-editing site, the authors may functionally discuss the host genes of these editing events.

** Following the reviewer comment, we added a sentence referring to the correlated genes (p. 8, 1st paragraph).

3. Although the authors are preparing a separated paper to explore differentially expressed genes, it would be better to perform PCA and examine whether the expression profile for the host genes of the editing sites in Fig. 4A can distinguish between the tasks of nurses and foragers.

** Given that the Editor has asked us to shorten the text, and that we prepare anyway another paper reporting the expression data, we do not think that we can fit this in (although we agree with the Reviewer that this information may be interesting). Adding the requested information requires substantial additional text for the Methods, Results, and Discussion that we believe adds only little to the current paper.

4. Regarding Drosophila RNA editing in Introduction and "conserved recoding editing sites" in Method, some related studies can be cited, e.g., Cell Reports 11(5), 2015, 697-703; Genome Biology and Evolution 10(2), 2018, 521-537; and PNAS, 2019, pnas.1811768116.

A. Cell Reports 11(5), 2015, 697-703 -- editing levels is affected by differences in cis-sequences between different drosophila species.

B. Genome Biology and Evolution 10(2), 2018, 521-537 -- identification of editing across species (including drosophila)

C. PNAS, 2019, pnas.1811768116. -- Each neuronal population in drosophila has a distinct editing signature

** We thank the Reviewer for calling our attention to these three interesting papers. We added a citation to the third paper (PNAS 2019, ref 18 at p. 3). However, given that we already have a very long list of references, we did not include the other two suggested references that we think are more remotely relevant to our story. We can nevertheless add them if the Editor finds it essential.
